# Trisubstituted Alkenes as Valuable Building Blocks

**DOI:** 10.3390/molecules30163370

**Published:** 2025-08-13

**Authors:** Tomáš Tobrman, Václav Hron

**Affiliations:** Department of Organic Chemistry, University of Chemistry and Technology, Prague, Technická 5, 166 28 Prague, Czech Republic; hronv@vscht.cz

**Keywords:** trisubstituted alkenes, ozonolysis, cyclization, addition

## Abstract

The stereoselective synthesis of trisubstituted alkenes has become a key topic in modern organic chemistry. At the same time, trisubstituted alkenes also serve as valuable starting materials for a wide range of transformations. However, it remains unclear to what extent these alkenes are utilized in comparison to their mono- and disubstituted counterparts. This review aims to provide a comprehensive overview of fundamental transformations involving all-carbon-substituted trisubstituted alkenes. The first section focuses on additions of carbon, oxygen, and nitrogen nucleophiles, as well as halogenation and carboxylation reactions. The second part discusses oxidative cleavage processes, while the final section addresses the cyclization and cycloisomerization reactions of trisubstituted alkenes.

## 1. Introduction

**Scheme 1:**
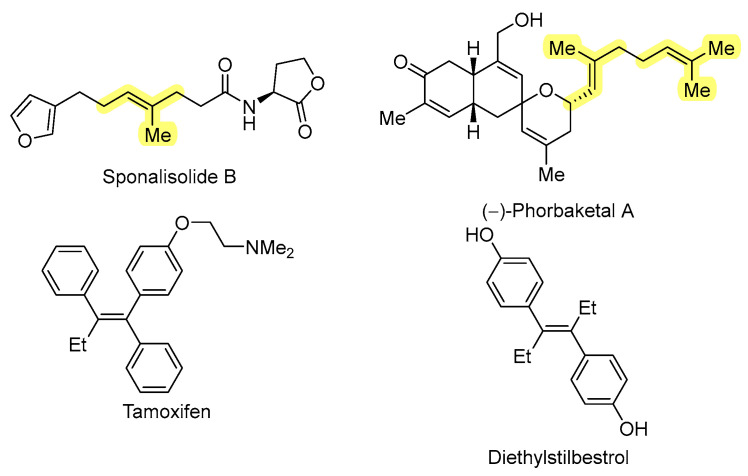
Naturally occurring trisubstituted alkenes.

The tetra- and trisubstituted double bonds represent a common motif found in natural products and medicinal substances. A typical example is the trisubstituted alkene sponalisolide B, which was isolated in racemic form from the sponge *Spongia officinalis* (Scheme 1). Sponalisolide B has been shown to function as a quorum sensing inhibitor in *Pseudomonas aeruginosa* [1]. Phorbaketal A, another example of a naturally occurring trisubstituted alkene, was isolated from the marine sponge *Phorbas* sp. and has demonstrated notable anti-inflammatory activity [2]. Among tetrasubstituted alkenes, tamoxifen is widely used in the treatment of breast cancer [3]. Another example is diethylstilbestrol (DES), a synthetic estrogen associated with numerous side effects [4]. Tri- and tetrasubstituted alkenes are widely employed in materials chemistry, with applications ranging from sensors to optoelectronic devices [5,6,7].

**Scheme 2:**
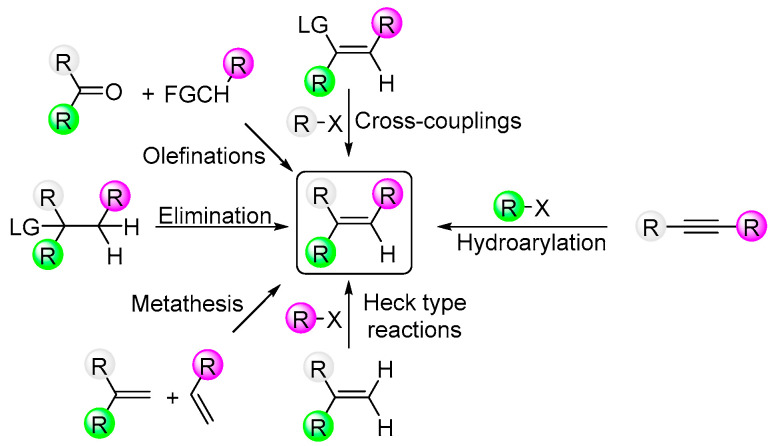
General scheme illustrating typical approaches to the stereoselective synthesis of trisubstituted alkenes.

The notable biological activities of tetra- and trisubstituted alkenes have driven the intensive development of stereoselective synthetic methods for their preparation. In principle, trisubstituted alkenes can be synthesized through cross-coupling reactions between electrophilic and nucleophilic reagents, such as the Heck [8,9,10,11] and alkyne hydroarylation reactions (Scheme 2) [12,13,14,15,16]. Alternatively, olefination and metathesis [17,18] reactions can be employed. Olefination methods in this context include various transformations, such as Julia [19,20,21,22,23], Still–Gennari [24], Wittig [25,26,27] and Horner–Wadsworth–Emmons olefination [28,29,30]. Comparable methods can be employed for the stereoselective synthesis of tetrasubstituted alkenes [31,32,33,34,35,36,37,38].

A number of papers have already been published in the field of stereoselective synthesis of trisubstituted alkenes. However, an important question remains: what is the use of trisubstituted alkenes in organic synthesis? Therefore, in this review, we sought to answer this seemingly simple question. This review article summarizes the important applications of all-carbon trisubstituted alkenes in organic synthesis, with a focus on studies published between 2015 and 2025. First, the addition of C-, O-, and N-nucleophiles to trisubstituted alkenes is discussed. This is followed by the oxidative cleavage of trisubstituted alkenes, and the last section discusses the cycloisomerization and cyclizations of trisubstituted alkenes. Because most of the cited studies include not only trisubstituted alkenes but also di- and tetrasubstituted ones, the overview indicates how many of the tested compounds are trisubstituted alkenes.

## 2. Results and Discussion

### 2.1. Addition Reactions

**Scheme 3:**
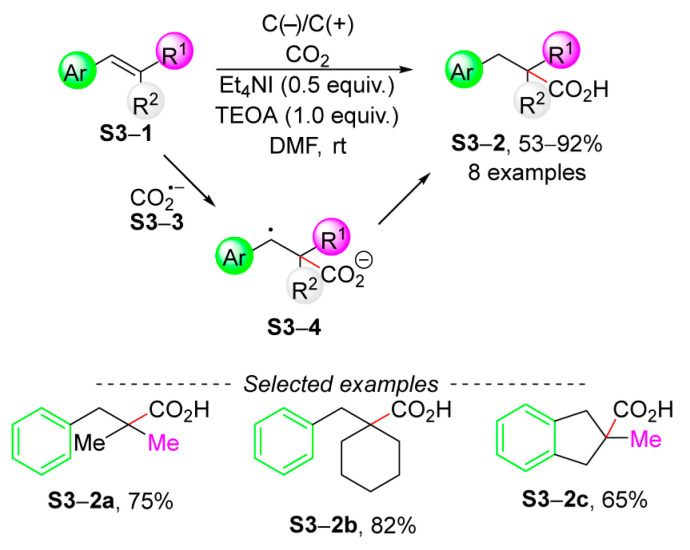
Electrochemical hydroxarboxylation of trisubstituted alkenes.

In 2020, Buckley reported a highly regioselective hydrocarboxylation method for the synthesis of alkyl carboxylic acids **S3–2** from trisubstituted alkenes (Scheme 3) [39]. The proposed mechanism involves the addition of a radical anion **S3–3** to the alkene, generating the most stable radical **S3–4**. A relatively broad substrate scope, encompassing 25 alkenes, was explored, including eight examples of trisubstituted alkenes. The corresponding alkyl carboxylic acids were obtained in satisfactory yields as shown by the selected examples.

**Scheme 4:**
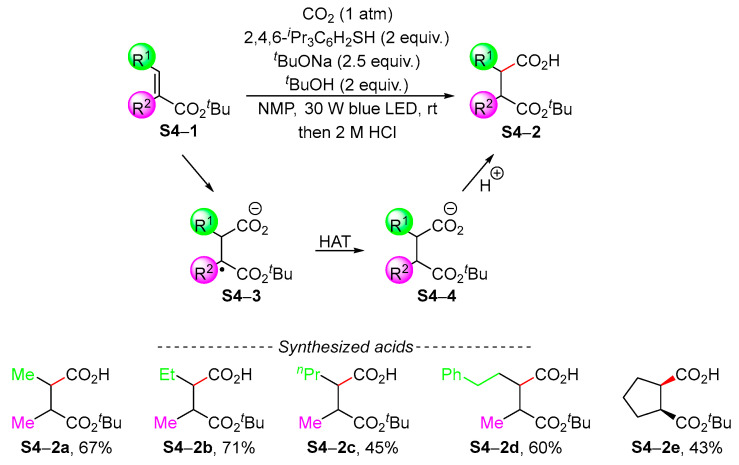
Transition metal-free carboxylation of acrylates.

Direct carboxylation of alkenes has also been reported in terms of substrate scope (Scheme 4) [40]. Among the total number of alkenes tested, five were trisubstituted acrylates. The structures of the corresponding products **S4–2a**–**S4–2e** are shown in Scheme 4. Based on quantum chemical calculations and experimental data, the proposed mechanism involves the formation of a stable radical species **S4–3**, which is subsequently converted into the final product via a hydrogen atom transfer (HAT) process followed by protonation.

**Scheme 5:**
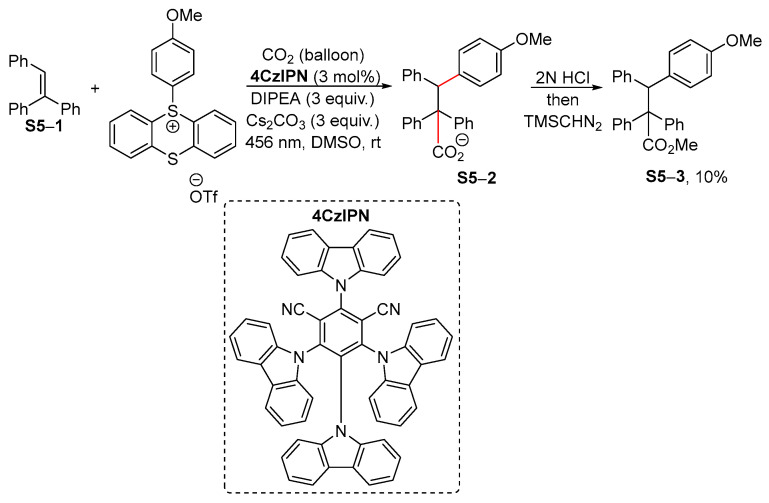
Carboxylative arylation of triphenyl ethylene.

Recently, Xie co-workers reported a carboxylation–alkylation reaction using thianthrenium salts and carbon dioxide (Scheme 5) [41]. Of the 41 products, only a single product **S5–3** was prepared from the trisubstituted alkene **S5–1**, with a 10% isolated yield. The primary product of the reaction between triphenyl ethylene and thianthrenium salt **S5–2** is a carboxylate salt, which is subsequently converted into an ester **S5–3** using trimethylsilyldiazomethane.

**Scheme 6:**
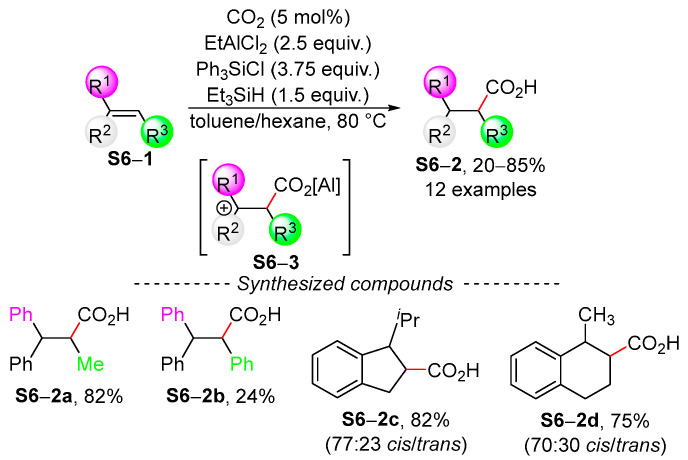
Hydrocarboxylation of trisubstituted alkenes.

Hydroxycarboxylation of alkenes was reported by Hattori and Tanaka (Scheme 6) [42]. The successful progression of the reaction requires the presence of dichloroethyl aluminum in combination with triethyl silane. Triethyl silane serves as a hydride ion source, which reacts with the in situ generated carbocation **S6–3** to form carboxylic acids **S6–2**. The reaction scope primarily focuses on trisubstituted alkenes, with 12 examples tested, but also includes a limited number of tetra- and disubstituted alkenes. For cyclic substrates, the reaction predominantly yielded the *cis* stereoisomer, as illustrated by examples **S6–2c** and **S6–2d**.

**Scheme 7:**
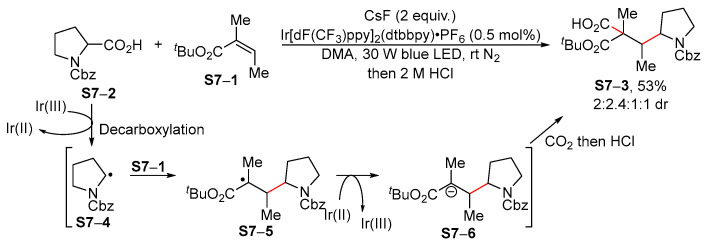
Photocatalytic iridium-catalyzed carbocarboxylation of a trisubstituted alkene.

Photocatalytic carbocarboxylation of trisubstituted alkenes was reported by Da-Gang Yu in 2021 (Scheme 7) [43]. This catalytic transformation converted alkene **S7–1** and amino acid **S7–2** into carboxylic acid derivative **S7–3** using an iridium complex under blue LED irradiation. Despite the broad substrate scope, which includes over 40 alkenes—primarily 1,1-disubstituted derivatives, only a single trisubstituted alkene **S7–1** was tested. The proposed mechanism involves the initial decarboxylation of the amino acid **S7–2** to generate a radical species **S7–4**, which adds to the alkene **S7–1** to form a new radical **S7–5,** which is reduced to anion **S7–6**. The light-activated iridium catalyst facilitates both the decarboxylation step and a radical–polar crossover process essential for product formation.

**Scheme 8:**
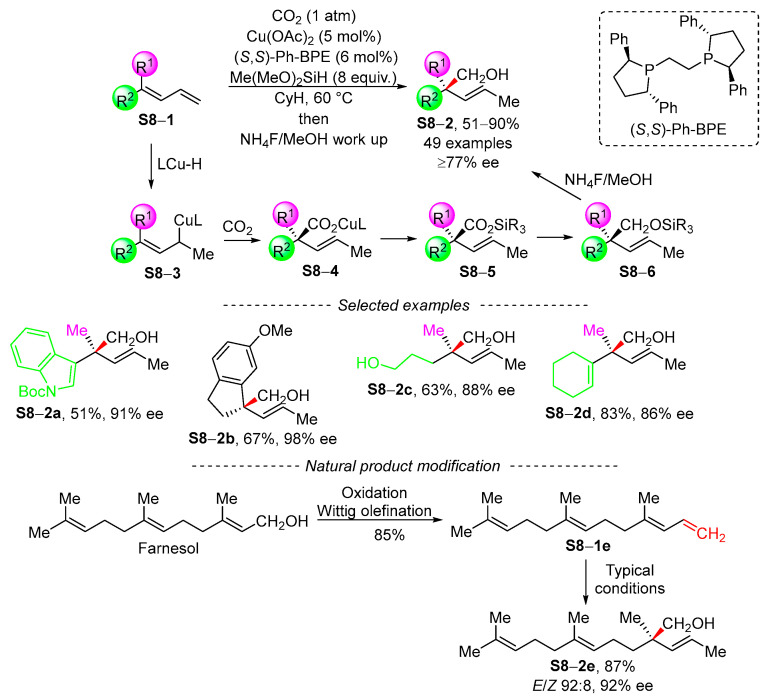
Copper-catalyzed hydroxymethylation of conjugated dienes.

Recently, a copper-catalyzed asymmetric hydroxymethylation of 1,3-dienes was reported (Scheme 8) [44]. The reaction employed copper acetate in the presence of eight equivalents of silane and a chiral ligand, delivering high levels of enantioselectivity. This transformation exhibited a broad substrate scope, covering 49 exclusively trisubstituted alkenes. A key feature of the methodology is its excellent enantioselectivity, which exceeds 90% ee in most cases, with only a few exceptions displaying lower values. Mechanistically, the transformation proceeds via the formation of a Cu–H hydride species, which undergoes hydrocupration with the starting alkene **S8–1**, generating an organocopper intermediate. Subsequent carboxylation yields carboxylic acid derivatives **S8–4** and **S8–5**, which are then reduced to the corresponding silylated alcohols. Final desilylation furnishes the hydroxymethylated product **S8–2**. The developed method was further applied to the derivatization of terpenoid compounds. In one representative example, Farnesol was first oxidized and converted into the diene **S8–1e** via a Wittig reaction. Subsequent hydroxymethylation afforded the product **S8–2e** in high yield and with excellent enantioselectivity.

**Scheme 9:**
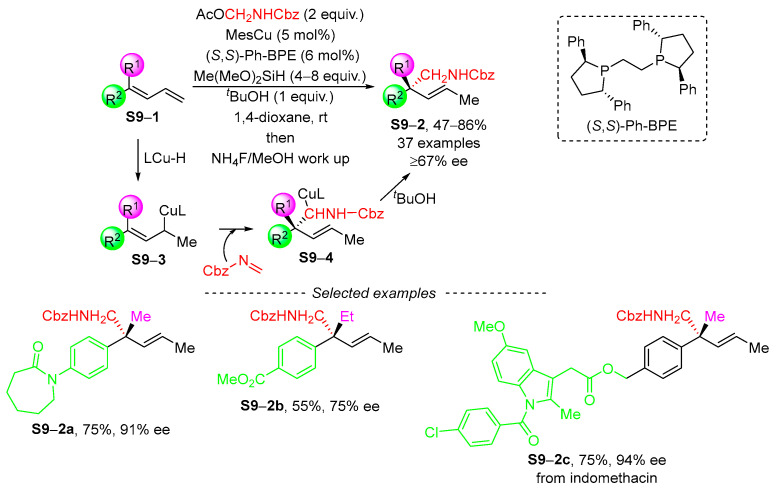
Copper-catalyzed aminomethylation of conjugated dienes.

**Scheme 10:**
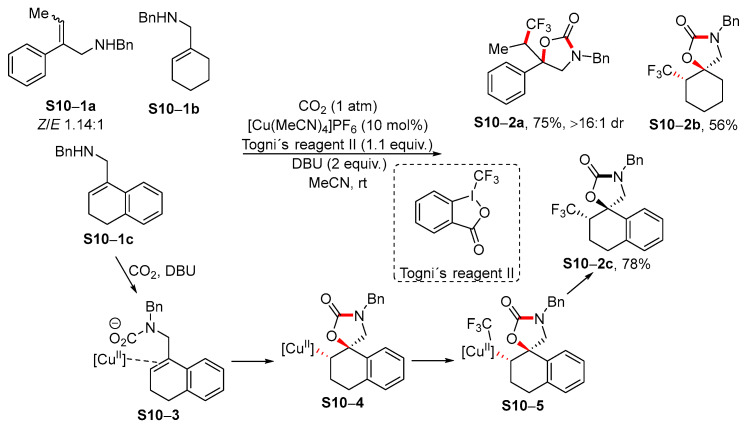
Copper-catalyzed carboxylative oxytrifluoromethylation of allylamines.

The developed methodology was also applied to the aminomethylation of trisubstituted alkenes (Scheme 9) [45]. Mechanistically, the reaction proceeds analogously to the previously described hydroxymethylation (Scheme 10). Specifically, the hydrocupration of diene **S9–1** generates the allylcopper intermediate **S9–3**, which reacts with an imine to form complex **S9–4**. Subsequent ligand substitution then yields the aminomethylated product **S9–2**. This transformation is characterized by a broad substrate scope, high enantioselectivity, and tolerance to highly functionalized trisubstituted alkenes, including an indomethacin-derived substrate **S9–2c**.

The divalent copper complex was also employed in the carboxylative oxytrifluoromethylation of allylamines. The reaction is conducted under a carbon dioxide atmosphere in the presence of Togni’s reagent and DBU, affording cyclic carbamates **S10–2** as the final products (Scheme 10) [46]. The substrate scope was evaluated using 24 alkenes, of which only three were trisubstituted. The starting alkene **S10–1a** was used as a mixture of *Z*/*E* isomers. In other tested cases, the reaction conditions proved tolerant of various functional groups, including nitro, ester, and amide moieties. A mechanism was proposed in which carbon dioxide is first activated via reaction with the allylamine **S10–1c**. The activated double bond then undergoes an intramolecular carboxylation, and subsequent trifluoromethylation along with reductive elimination converts the Cu(II) complex **S10–3** into the final product **S10–2c**. Following the carboxylative trifluoromethylation, the formation of cyclic carbamates via the alkylative carboxylation of allylamines has also been reported. This transformation proceeds under visible-light irradiation using a catalytic amount of a palladium complex. However, the substrate scope is limited, with only a single example involving a trisubstituted alkene [47].

**Scheme 11:**
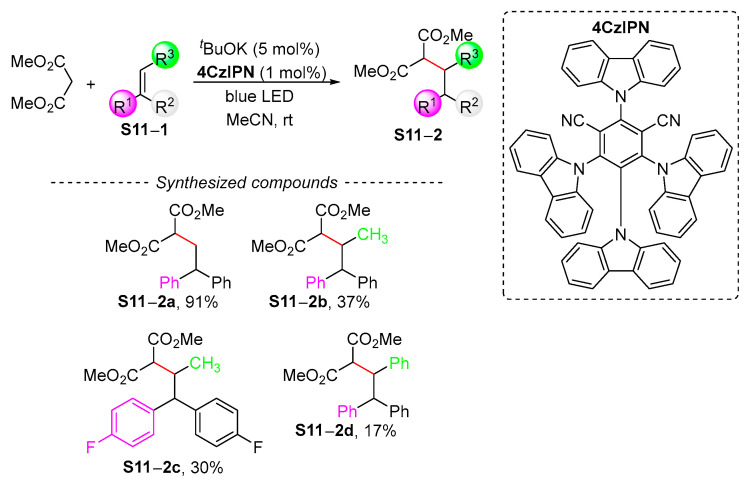
Photocatalyzed addition of dimethyl malonate to trisubstituted alkenes.

In 2020, Kobayashi published a study on the photocatalyzed addition of malonates to tri- and disubstituted alkenes (Scheme 11) [48]. The reaction was catalyzed using a carbazole-based photocatalyst. Among the 35 alkenes tested, only 3 examples of trisubstituted alkenes were explored, yielding malonates **S11–2b**–**S11–2d**. Notably, 1,1-disubstituted alkenes provided significantly higher yields, as demonstrated by the synthesis of derivative **S11–2a**. From a mechanistic perspective, the authors proposed that the photocatalyst oxidizes the malonate to its corresponding radical, which subsequently adds to the starting alkene.

**Scheme 12:**
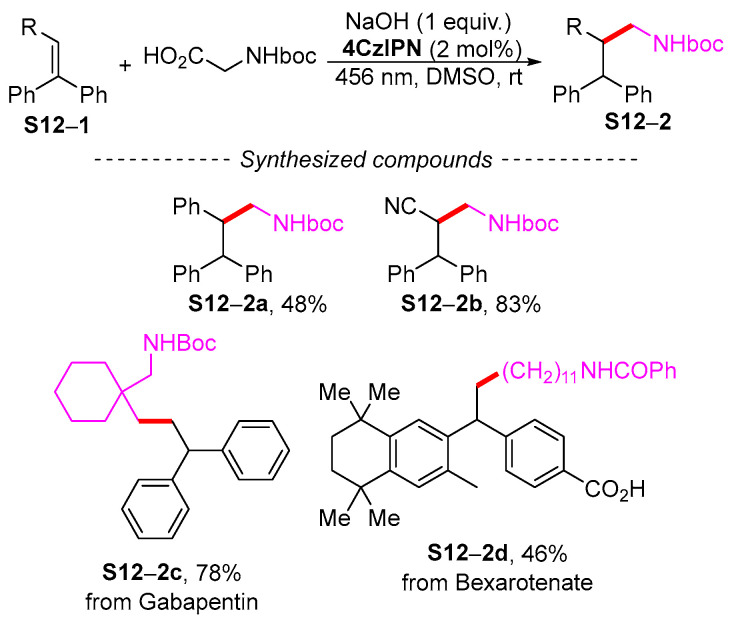
Photocatalyzed hydroaminomethylation of trisubstituted alkenes.

Similarly to Kobayashi, Das described the photocatalyzed hydroaminomethylation of primarily disubstituted alkenes using the same photocatalyst (Scheme 12) [49]. Out of 60 examples, only 2 amino derivatives, **S12–2a** and **S12–2b**, were synthesized from trisubstituted alkenes. In the remaining cases, only di- and monosubstituted alkenes were used. Like Kobayashi, Das proposed a radical mechanism for the formation of the target alkenes **S12–2**, which is supported by quantum chemical calculations. The optimized reaction conditions were subsequently applied to the synthesis of pharmaceutical derivatives, such as **S12–2c** and **S12–2d**. However, only mono- and disubstituted alkenes were used in these examples.

**Scheme 13:**
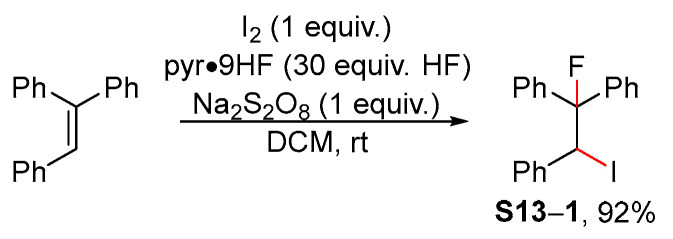
Transition-metal-free iodofluorination of triphenylethylene.

Trisubstituted alkenes can also participate in halogen addition reactions. A representative example is the synthesis of 2-fluoroalkyl iodides via the reaction of an alkene with iodine in the presence of hydrofluoric acid and an oxidizing agent (Scheme 13) [50]. The optimized reaction conditions were tested on a total of 15 alkenes; however, only one of the tested substrates was a trisubstituted alkene. The corresponding product, **S13–1**, was obtained in nearly quantitative isolated yield. According to the authors, the formation of **S13–1** proceeds via the in situ transformation of I_2_ into IF, which subsequently adds to the double bond.

**Scheme 14:**
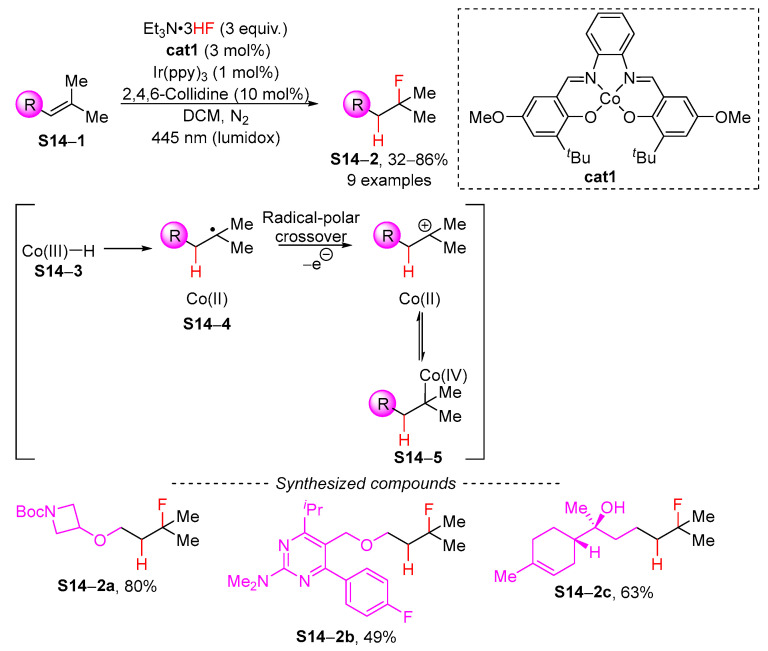
Cobalt-catalyzed hydrofluorination of trisubstituted alkenes.

Recently, a cobalt-catalyzed hydrofluorination of alkenes, including trisubstituted examples, was reported. The reaction employs the cobalt–salen complex **Cat1**, which plays a key role in the formation of the target compounds (Scheme 14) [51]. Initially, the cobalt complex is converted into the hydride species **S14–3**, which reacts with isoprenyl alkenes to generate the stabilized radical intermediate **S14–4**. A subsequent radical–polar crossover leads to the formation of the Co(IV) complex **S14–5**, which undergoes nucleophilic attack by a fluoride anion. The involvement of a carbocationic intermediate was supported by carbocation rearrangement experiments. This methodology features a broad substrate scope, encompassing 31 alkenes, including 9 trisubstituted ones. The reaction exhibits excellent functional group tolerance; however, sensitive groups such as aldehydes and ketones were not tested. Notably, the process demonstrates high chemoselectivity, as illustrated by substrates bearing multiple unsaturations **S14–2c**. In addition, the method allows for the efficient synthesis of fluorinated products incorporating ^19^F atoms.

**Scheme 15:**
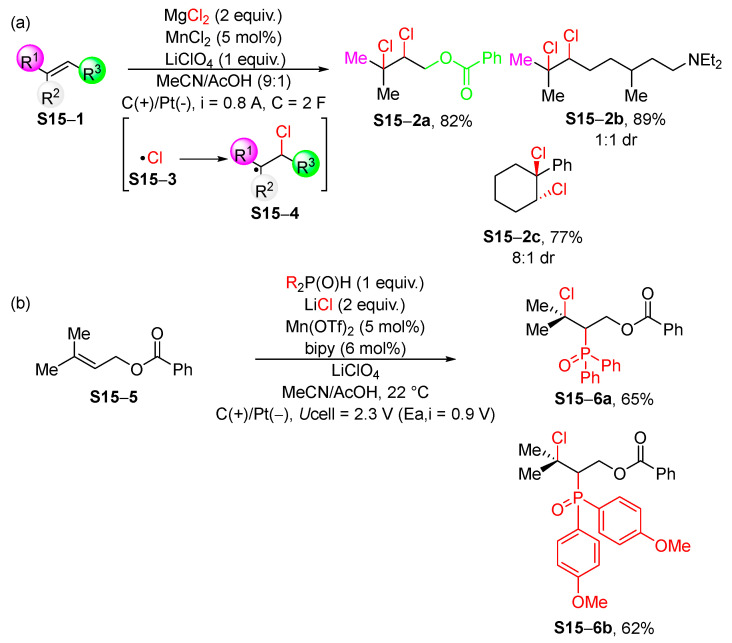
(**a**) Electrocatalytic chlorination and (**b**) electrocatalytic chlorophosphination of trisubstituted alkene.

Electrocatalytic transformations have been employed for the straightforward dichlorination of trisubstituted alkenes using a nucleophilic chlorine source (Scheme 15a) [52]. Among the 26 substrates tested, only three were trisubstituted alkenes: **S15–2a**, **S15–2b,** and **S15–2c**. A gram-scale reaction was demonstrated using indene, which proceeded in high yield and with excellent diastereoselectivity. The proposed mechanism mirrors that of the previously described transformation and is supported by experimental evidence, including cyclic voltammetry data. An extension of the previously reported electrochemical dichlorination is its implementation in the presence of diarylphosphine oxide (Scheme 15b) [53]. In this variant, lithium chloride serves as the chloride source for the generation of Cl•, and the reaction is catalyzed by manganese triflate. Unfortunately, the substrate scope is limited, with only 1 trisubstituted alkene **S15–5** successfully converted out of the 16 alkenes tested.

**Scheme 16:**
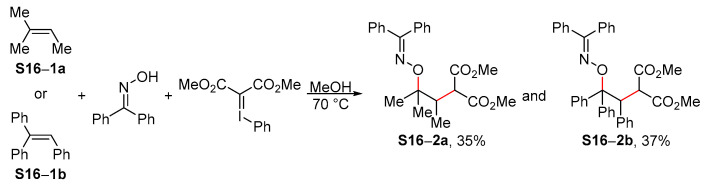
Addition of an iodonium ylide to trisubstituted alkenes.

Another example of the transformation of trisubstituted alkenes limited in scope is the addition of iodonium ylides (Scheme 16) [54]. The authors optimized the reaction conditions to modify a wide range of alkenes, covering over 35 examples; however, only 2 alkenes, **S16–1a** and **S16–1b**, were trisubstituted. The limited use of trisubstituted alkenes may be due to the relatively low yields obtained for products **S16–2a** and **S16–2b**. Most reactive disubstituted alkenes afforded products in yields ranging from 50% to 80%.

**Scheme 17:**
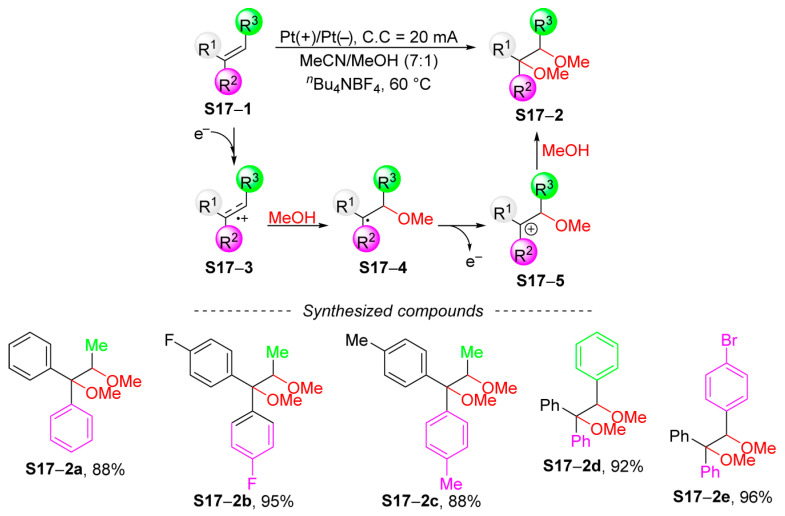
Electrochemical dimethoxylation of trisubstituted alkenes.

Xu et al. reported an electrochemical dimethoxylation of alkenes with reaction conditions optimized for 1,1-disubstituted ethylenes (Scheme 17) [55]. However, these optimized conditions were successfully extended to trisubstituted alkenes **S17–1**, leading to the formation of five dimethoxy derivatives **S17–2a**–**S17–2e**. The proposed mechanism suggests that the starting alkene is first oxidized at the anode to generate radical cation **S17–3**, which subsequently reacts with methanol to form radical **S17–4**. Further oxidation converts this radical into carbocation **S17–5**, which ultimately yields the main reaction product **S17–2**.

**Scheme 18:**
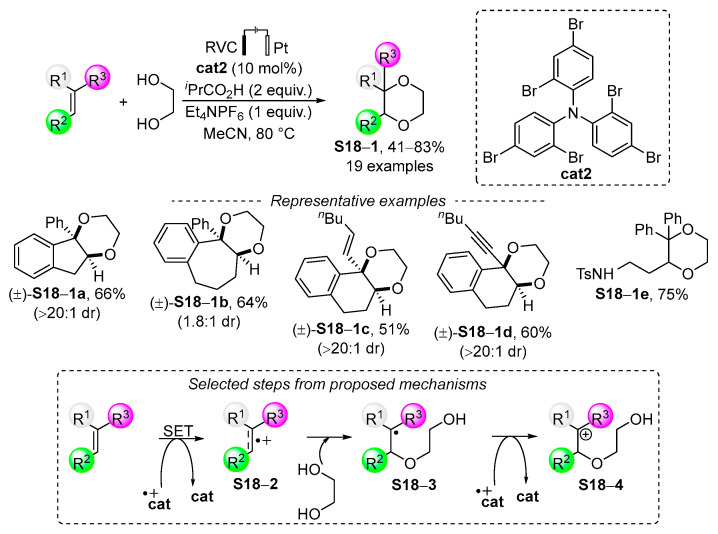
Electrochemical transformation of trisubstituted alkenes into 1,4-dioxane derivatives.

Among the transition metal–free approaches to the functionalization of trisubstituted alkenes, electrochemical dihydroxylation represents a noteworthy example (Scheme 18) [56]. The reaction employs a reticulated vitreous carbon (RVC) anode and a platinum cathode, and requires the presence of a redox mediator. In this context, triarylamine was identified as the optimal redox catalyst (**cat2**). The optimized conditions proved to be broadly applicable, as demonstrated by successful transformation of 19 trisubstituted alkenes out of a total of 37 substrates tested. The reaction displays excellent diastereoselectivity in the case of five- and six-membered cycloalkenes **S18–1a** and **S18–1c**; however, the corresponding cycloheptene derivative **S18–1b** afforded a product with markedly reduced diastereoselectivity. The proposed mechanism involves the initial formation of a radical cation intermediate, which undergoes further transformation to a stabilized carbon-centered radical **S18–3**. Subsequent single-electron oxidation generates a carbocation intermediate **S18–4**, which then undergoes nucleophilic attack to afford the final dihydroxylated product **S18–1**.

**Scheme 19:**
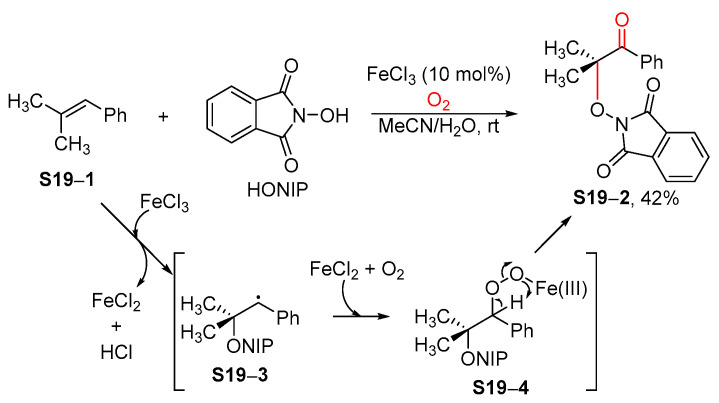
Iron(III)-catalyzed hydroxy-phthalimidation of (2-methylprop-1-en-1-yl)benzene.

Further radical functionalization of alkenes and styrenes was reported by Tang in 2016 (Scheme 19) [57]. In this case, the oxidation is catalyzed by ferric chloride in the presence of oxygen, leading to the formation of α-substituted ketones. More than 30 alkenes have been oxidized using this method, but only one trisubstituted alkene **S19–1** was tested. Thus, the oxidation of alkene **S19–1** afforded ketone **S19–2** in a 42% isolated yield. The proposed mechanism suggests that ferric chloride catalyzes the formation of the •ONPI radical, which adds to the starting alkene, generating the stable radical **S19–3**. This radical is then oxidized by oxygen in the presence of ferric chloride to form peroxide **S19–4**, which undergoes further rearrangement to yield ketone **S19–2**.

**Scheme 20:**
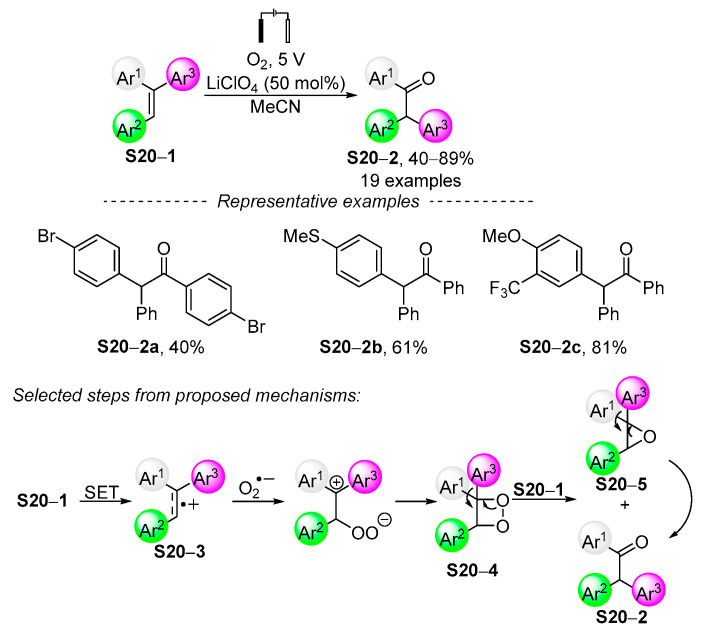
Electrochemical oxygenation of trisubstituted alkenes.

A surprisingly broad electrochemical oxygenation of trisubstituted alkenes has been reported recently (Scheme 20) [58]. The transformation is performed in an undivided electrochemical cell equipped with two graphite felt electrodes. The methodology was evaluated exclusively on trisubstituted alkenes and delivered excellent yields of the corresponding ketones **S20–2**. However, the substrate scope is notably limited to triaryl alkenes bearing halogen, alkylthio, or alkoxy substituents. As is characteristic of electrochemical processes, the reaction proceeds via formation of the radical cation intermediate **S20–3**, which undergoes coupling with a superoxide anion to generate the peroxy radical intermediate **S20–4** after cyclization, which undergoes disproportionation to afford the desired ketone **S20–2** product along with epoxide **S20–5** as a side product.

**Scheme 21:**
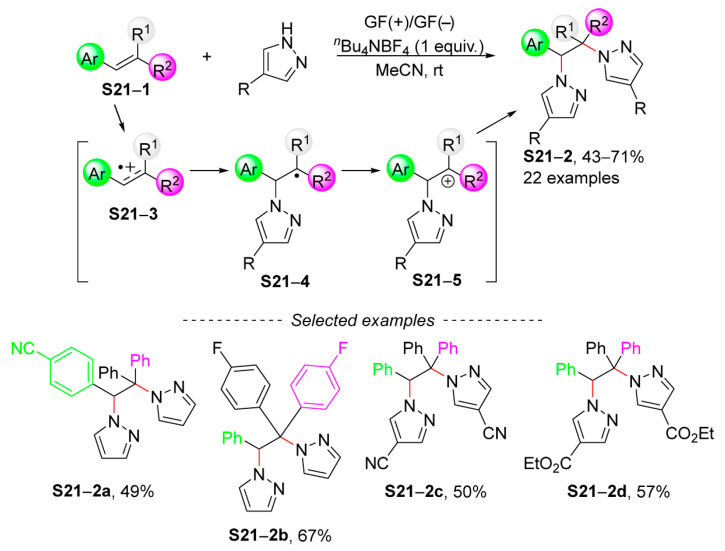
Electrochemical diazolation of trisubstituted alkenes.

The electrochemical addition of pyrazoles to trisubstituted alkenes was reported in 2023 (Scheme 21) [59]. The reaction is generally limited in scope to trisubstituted alkenes, with only a single example involving a tetrasubstituted alkene. In contrast, disubstituted alkenes were found to be unreactive under the reported conditions. This lack of reactivity may be attributed to the difficulty of anodically oxidizing disubstituted alkenes to the corresponding radical cations **S21–3**. Upon a second anodic oxidation, the initially formed radical **S21–4** is converted into a carbocation **S21–5**, which subsequently reacts with pyrazole to afford the final product **S21–2**. The reaction exhibits good functional group tolerance, as substrates bearing ester or nitrile substituents were successfully employed.

**Scheme 22:**
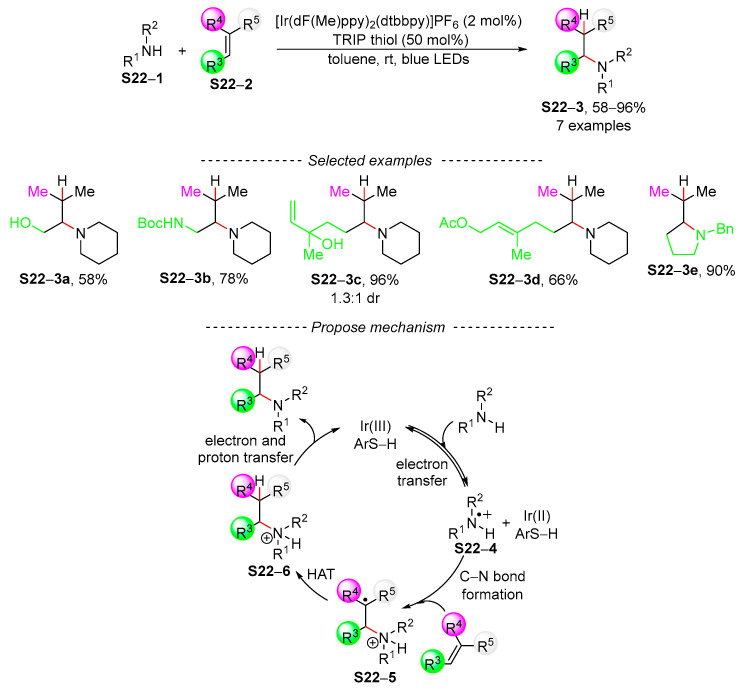
Photocatalytic hydroamination of trisubstituted alkenes.

Photoredox catalysis has also been employed for the hydroamination of trisubstituted alkenes. A reaction between secondary amines and alkenes, catalyzed by an iridium complex, was reported by Knowles and co-workers (Scheme 22) [60]. The transformation requires the presence of 2,4,6-triisopropylbenzenethiol and a catalytic amount of [Ir(dF(Me)ppy)_2_(dtbbpy)]PF_6_. The proposed mechanism involves the formation of a radical cation **S22–4** alongside the Ir(II) species. This radical cation subsequently reacts with the alkene **S22–2** to generate the carbon-centered radical **S22–5**. Hydrogen atom transfer (HAT) then yields the ammonium intermediate **S22–6**, which undergoes proton and electron transfer to afford the final tertiary amine product **S22–3**. Nevertheless, the substrate scope remains limited for trisubstituted alkenes, with only 7 examples reported out of the 50 tested. On the other hand, the reaction tolerates a variety of functional groups, including esters, amides, and hydroxyl groups.

**Scheme 23:**
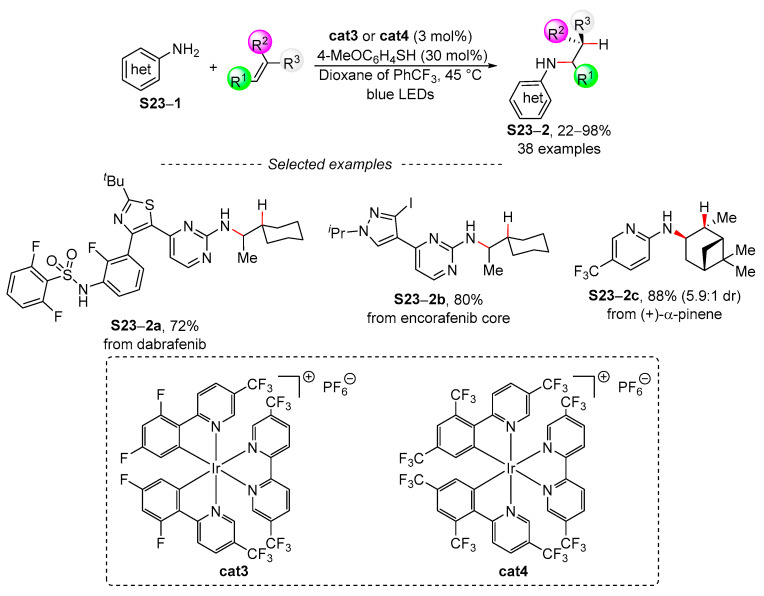
Hydroamination of trisubstituted alkenes using primary heteroaromatic amines.

A significantly broader substrate scope in terms of trisubstituted alkene structures was reported for the photoredox-catalyzed hydroamination of trisubstituted alkenes (Scheme 23) [61]. In this reaction, amines bearing heteroaromatic substituents are added in an anti-Markovnikov fashion. The transformation displays excellent substrate tolerance, with 38 out of 54 tested alkenes falling into the trisubstituted category. Despite the broad substrate scope, the structural diversity is largely limited to nitrogen-containing heterocyclic systems. The tolerated functional groups are mostly robust; notably, highly reactive functionalities such as aldehydes and ketones are absent. The methodology was also successfully applied to biologically relevant molecules, as illustrated by the selected examples **S23–2a**, **S23–2b**, and **S23–2c**.

**Scheme 24:**
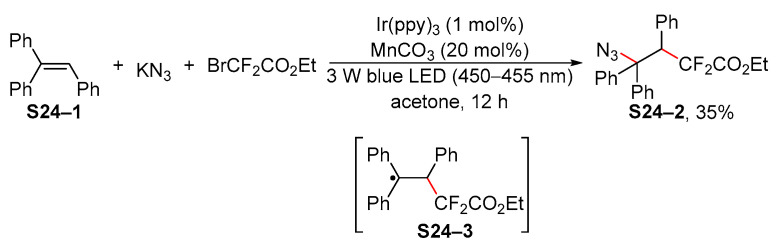
Photocatalyzed azidodifluoroalkylation of trisubstituted alkene.

A significant advancement in the field of radical addition to alkenes was reported by Meng and Ren (Scheme 24) [62]. The described reaction enables the direct azidodifluoroalkylation of triphenyl ethylene through photoredox synergistic catalysis. This synergistic effect was achieved using an iridium–manganese catalytic system. In this process, the iridium complex generates a •CF_2_CO_2_Et radical, which adds to the starting alkene **S24–1**. The presence of manganese salts facilitates the azidation of the carbon-centered radical **S24–3** via the formation of a Mn(III)–N_3_ intermediate. Although the reaction demonstrates excellent scope, covering 25 alkenes, triphenyl ethylene was the only trisubstituted alkene utilized.

**Scheme 25:**
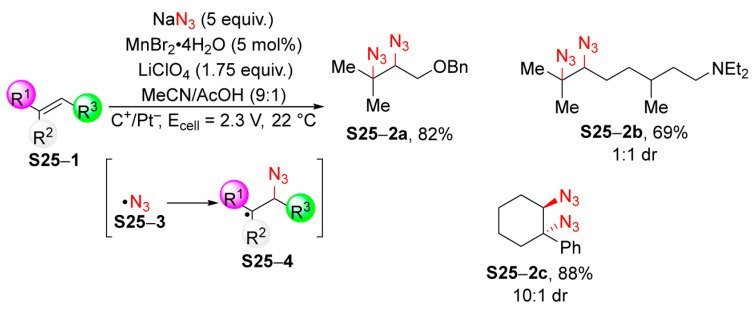
Electrocatalytic diazidation and dichloration of trisubstituted alkenes.

Electrochemical transformations of trisubstituted alkenes have also been applied to diazidation reactions. The transformation employs a simple electrochemical setup and requires only a catalytic amount of manganese bromide (Scheme 25) [63,64]. Mechanistic studies suggest that sodium azide is electrochemically reduced to the azidyl radical **S25–3**, which adds to the starting alkene to form the radical intermediate **S25–4**. Unfortunately, the scope of trisubstituted alkenes tested in this transformation is very limited. Out of more than 50 alkenes evaluated, only 3 trisubstituted alkenes were included, affording the products **S25–2a**, **S25–2b**, and **S25–2c**. In contrast to disubstituted alkenes, the trisubstituted examples exhibit not only a narrower substrate range, but also limited diversity in the functional groups tested.

**Scheme 26:**
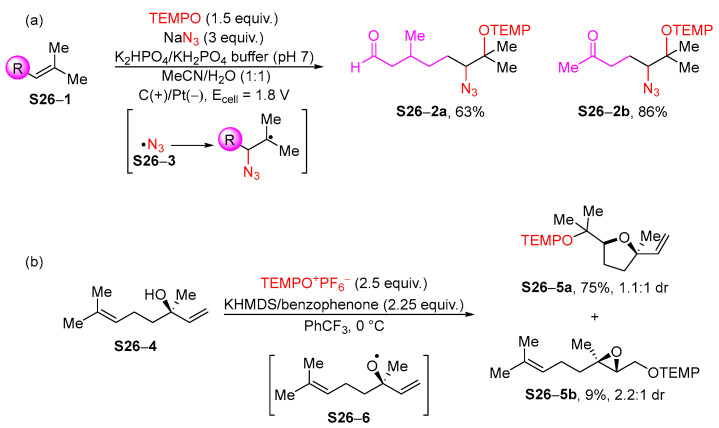
(**a**) Electrochemical azidooxygenation of trisubstituted alkenes and (**b**) cyclization of alkanols.

Electrochemical diaziridinization was further extended to the azidooxygenation of alkenes, including trisubstituted alkenes, by Prof. Lin’s research group (Scheme 26a) [65]. With respect to trisubstituted alkenes, the scope of the methodology remains limited, as only 2 examples were included out of the 23 alkenes tested. However, a notable feature of this study is the successful application of substrates bearing sensitive functional groups such as the aldehydes and ketones **S26–2a** and **S26–2b**. TEMPO was also employed in the oxidative ring opening of cycloalkanols and in the cyclization of alkanols (Scheme 26b) [66]. The cyclization of tertiary alcohols was demonstrated using α-bisabolol and (*R*)-linalool (**S26–4**) as substrates. An interesting observation is that the cyclization of (*R*)-linalool (**S26–4**) proceeds predominantly via a 5-exo-trig pathway, affording **S26–5a** as the major product.

### 2.2. Oxidative Cleavage of Trisubstituted Alkenes

The oxidative cleavage of alkenes—commonly known as ozonolysis—is a fundamental transformation in organic synthesis. First discovered in 1840, it remains a widely employed strategy for diversity-oriented synthesis. A recent example involves the ozonolysis of alkenes in a continuous flow film-shear reactor (Table 1, entry 1) [67]. During the ozonolysis of triphenyl ethylene, the authors observed the formation of a mixture of benzophenone (**T1–1a**) and secondary ozonide **T1–1b** in a ratio of 3.4:1. In other cases, only mono- and disubstituted alkenes, along with a single example of a tetrasubstituted alkene, were tested. Although ozone is a powerful oxidizing agent, its application is limited by its inherent instability. Consequently, alternative methods are being explored to achieve efficient oxidative cleavage of C=C bonds under mild conditions using readily available reagents. One such option is the cleavage of C=C bonds using hydrogen peroxide, which offers a more accessible and stable alternative. A readily available option for C=C bond cleavage is the use of hydrogen peroxide. Yu reported a diselenide-catalyzed oxidation of alkenes (Table 1, entries 2 and 3). In the oxidation of alkenes catalyzed by (c-C_6_H_11_Se)_2_ in the presence of ferric nitrate, a proposed mechanism involves the epoxidation of the double bond to either epoxide **T1–2a** or peroxide **T1–2b**, depending on whether the reaction proceeds via an ionic or radical pathway (Table 1, entry 2) [68]. Similarly, the diselenide-catalyzed oxidation of C=C bonds can proceed without ferrous salts (Table 1, entry 3) [69]. The optimized reaction conditions were explored across a wide range of trisubstituted alkenes. The proposed oxidation mechanism involves the initial formation of an epoxide **T1–2a** from the alkene, which is subsequently converted into peroxoester **T1–3a**. Fan reported an oxidative cleavage of the C=C bond catalyzed by an Fe(II) complex (**Cat5**) in the presence of excess hydrogen peroxide (Table 1, entry 4) [70]. The scope of the optimized reaction conditions was limited to only three trisubstituted alkenes, including one bearing a nitrile group. The authors proposed that the oxidation of the alkenes proceeds via the peroxo intermediate **T1–4a**. The universal ruthenium complex (**Cat2**) has been employed in the oxygenation of alkynes, amidation of aldehydes, and oxidative cleavage of alkenes (Table 1, entry 5) [71]. In these transformations, sodium iodate serves as the terminal oxidant, converting the precatalyst (**Cat6**) to a Ru^IV^O_2_ species. A subsequent [3+2] cycloaddition between this complex and the alkene furnishes a Ru(IV) intermediate (**T1–5a**), which undergoes fragmentation to afford the final oxidation products. However, the optimized reaction conditions were applied to only a single example of a trisubstituted alkene.

An alternative method for oxidizing alkenes to carbonyl compounds involves the use of oxygen in the presence of various catalysts. Zhou (2021) reported the oxidation of primarily disubstituted alkenes by oxygen in poly(ethylene glycol) dimethyl ether (PEGDME) (Table 2, entry 1) [72]. Of the wide range of alkenes tested, only two trisubstituted alkenes were used, which were oxidized to benzophenone (**T1-1a**) and benzaldehyde (**T1-2c**) in nearly quantitative yields. The authors proposed that the formation of the peroxide intermediate **T2-1b** is a key step in the oxidation of the C=C bond. The formation of peroxide **T2-1b** is facilitated by the oxidation of PEGDME by oxygen to peroxide **T2-1a**. Additionally, sodium benzenesulfinate can be used for the photochemical oxidation of trisubstituted double bonds, although the scope of the reaction is limited to a single case (Table 2, entry 2) [73]. Based on the typical reactivity of sulfinic acid salts, it has been proposed that the addition of the PhSO_2_• radical produces an intermediate **T2–2a**, which undergoes nucleophilic substitution. The resulting cyclic peroxide then decomposes into oxidation products **T1–2c** and **T2–2b**. Photoinduced oxidation of alkenes was reported by Parasram (Table 2, entry 3) [74]. The optimized reaction conditions cover a wide range of alkenes, including 1,1-disubstituted, monosubstituted, and trisubstituted alkenes. The tolerance of functional groups is relatively broad, with ester, keto groups, and halogens being tolerated. The authors of the paper also suggested that cleavage of the C=C bond involves the formation of the cyclic intermediate **T2–3a**. A photochemical oxidative cleavage of di-, tri-, and tetrasubstituted alkenes was reported in 2021 (Table 2, entry 4) [75]. The transformation is catalyzed by a manganate complex under an oxygen atmosphere. Mechanistic studies suggest that photoexcitation promotes oxidation of the manganese complex to an Mn(III) species, which then engages the alkene to generate a carbon-centered radical intermediate (radical **T2–4a**). Subsequent reaction with molecular oxygen affords a peroxo complex that undergoes decomposition to deliver the cleavage products. Notably, the reaction proceeds under mild conditions and avoids the use of conventional stoichiometric oxidants such as hydrogen peroxide or ozone. Unfortunately, out of a total of 80 alkenes tested, only 3 were trisubstituted alkenes.

**Scheme 27:**
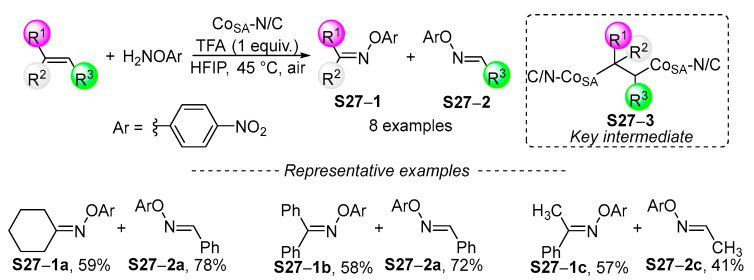
Cobalt-catalyzed oxidative cleavage of alkenes to oximes.

Unidative C=C bond grafting has also been demonstrated using a single-atom cobalt catalyst (Scheme 27) [76]. The catalyst (Co_SA_–N/C) was obtained via pyrolysis of a bimetal–organic framework (ZnCo-BMOF) and was subsequently employed for the direct transformation of alkenes into oximes. As illustrated in Scheme 19, the reaction conditions were applied to a set of 31 alkenes, including 8 trisubstituted substrates. The proposed mechanism involves the formation of an ArON• radical, which adds to the alkene double bond to afford an intermediate species **S27–3**. Subsequent cleavage of the C–C bond in this intermediate furnishes the corresponding oxime products.

**Scheme 28:**
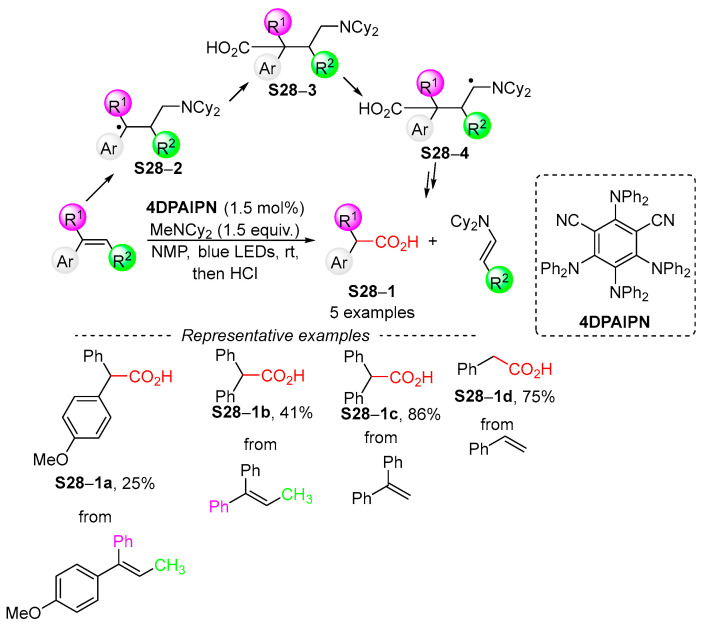
Photocatalytic cleavage of alkenes via CO_2_ incorporation.

Another example of photocatalytic carboxylative C=C bond grafting was reported in 2024 (Scheme 28) [77]. The reaction requires 1.5 equivalents of methyldicyclohexylamine, which undergoes single-electron transfer (SET) to generate an amine-derived radical. Addition of this radical to the alkene forms the most stable carbon-centered radical intermediate **S28–2**. Subsequent carboxylation with carbon dioxide yields the corresponding carboxylic acid **S28–3**. A second SET event, followed by elimination of the imine, furnishes the final product **S28–1**. Despite the broad substrate scope—exceeding 70 alkenes—only 5 trisubstituted alkenes were included.

### 2.3. Ring Formation Involving Trisubstituted Alkenes

In addition to undergoing oxidative or otherwise destructive modifications, trisubstituted alkenes can also participate in a wide range of cyclization and cycloisomerization reactions. These transformations may proceed either under transition metal catalysis or under metal-free conditions. A particularly well-explored class of such reactions involves cyclizations leading to indole derivatives, for which a variety of starting materials have been employed.

Aniline derivatives bearing trisubstituted double bonds are widely employed in the synthesis of indole derivatives. A particularly straightforward method involves NIS-induced cyclization of the starting aniline substrates. However, the reaction has been predominantly studied with disubstituted alkenes, with only four examples of trisubstituted alkenes reported. The proposed mechanism for the formation of indole derivatives involves NIS-mediated generation of the cationic intermediate **T3–1a**, which subsequently undergoes cyclization to form the iodinated intermediate **T3-1b**. The final products are obtained through hydrogen iodide elimination followed by aromatization. The formation of the cationic intermediate **T3–1a** was indirectly supported by the observation of an aryl shift in trisubstituted alkene **T3-1e** (Table 3, entry 1) [78]. A similar cyclization was reported by Youn and co-workers (Table 3, entry 2) [79]. However, unlike the previous approach, the cyclization of aniline derivatives in this case was mediated by silver carbonate in DMF. The proposed mechanism involves a single-electron transfer (SET) process along with a radical–polar crossover. The scope of the reaction was relatively limited, with only eight trisubstituted alkenes successfully undergoing cyclization out of the fifty alkenes tested. The same research group later reported an analogous cyclization coupled with the isomerization of aryl substituents. The reaction was carried out using palladium acetate (5 mol%) and cupric chloride (2.0 equivalents) in 1,2-dichloroethane at 150 °C [80]. The reaction scope was limited to only four trisubstituted alkenes out of a total of seventeen alkenes tested. The cyclization of aniline derivatives to indoles can also be catalyzed by p-toluenesulfonic acid (Table 3, entry 3) [81]. This reaction is remarkable in that it is performed in the presence of benzoquinone, which becomes incorporated into the indole framework. The presence of the benzoquinone moiety in the product was rationalized by a direct condensation between benzoquinone and aniline, forming intermediate **T3–3a**, which subsequently cyclizes to the carbocation **T3–3b**. Notably, the reaction also exhibits a distinctive scope, as it enables the synthesis of indole derivatives bearing the ester functional groups **T3–3c** and **T3–3d**.

**Scheme 29:**
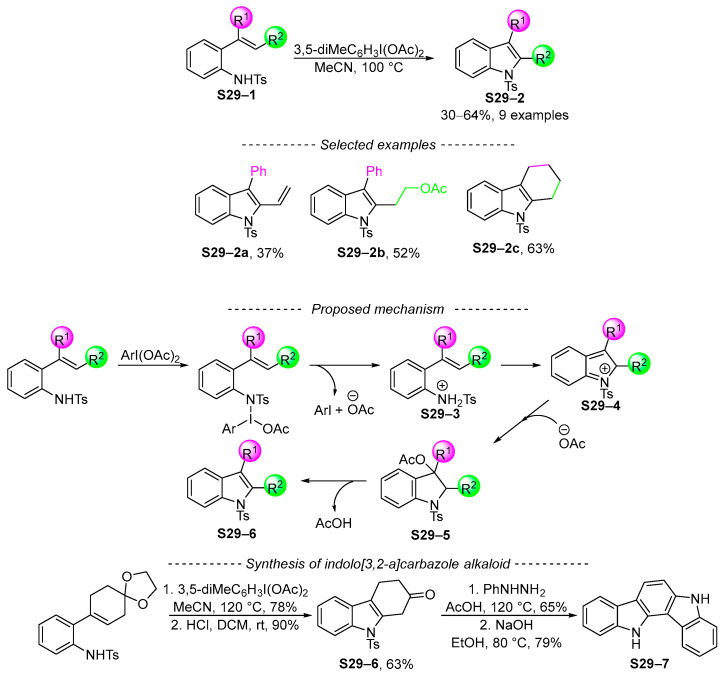
Iodine(III)-mediated intramolecular amination of a trisubstituted alkene for the synthesis of an indolo[3,2-a]carbazole alkaloid.

As an extension to previous cyclizations of ortho-substituted anilines, the iodine(III)-mediated cyclization of trisubstituted alkenes has been reported (Scheme 29) [82]. Through careful optimization of the reaction conditions, the authors found that the highest yields of substituted indoles **S29–2** were achieved using 3,5-dimethylphenyl-λ^3^-iodane [3,5-Me_2_C_6_H_3_I(OAc)_2_] in acetonitrile as the solvent. The reaction is notable for its short reaction time (approximately 20 min) and broad substrate scope, encompassing 18 geminally disubstituted alkenes and 9 trisubstituted alkenes. A mechanistic proposal involves the formation of cationic intermediates **S29–3** and **S29–4**, which is followed by the reaction with acetate and elimination to afford the desired products. This methodology was successfully applied to the synthesis of the carbazole alkaloid **S29–7**, which was obtained in a 36% isolated yield over four steps.

**Scheme 30:**
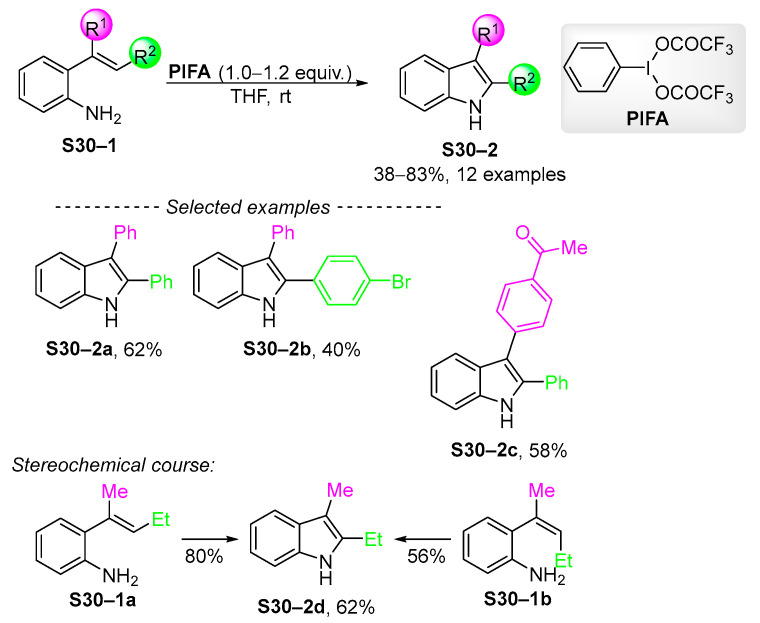
PIFA-mediated cyclization of trisubstituted alkenes to indole derivatives.

An exceptionally broad-scope cyclization of trisubstituted alkenes has been reported by Kim and Cha (Scheme 30) [83]. The reaction employs the iodine(III) reagent PIFA and proceeds smoothly at room temperature. A distinctive feature of this study is the exclusive use of trisubstituted alkenes **S30–1**, predominantly as mixtures of *E*/*Z* isomers. A detailed investigation of the reaction course revealed that (*E*)-alkenes afford higher yields of disubstituted indoles compared to (*Z*)-alkenes.

**Scheme 31:**
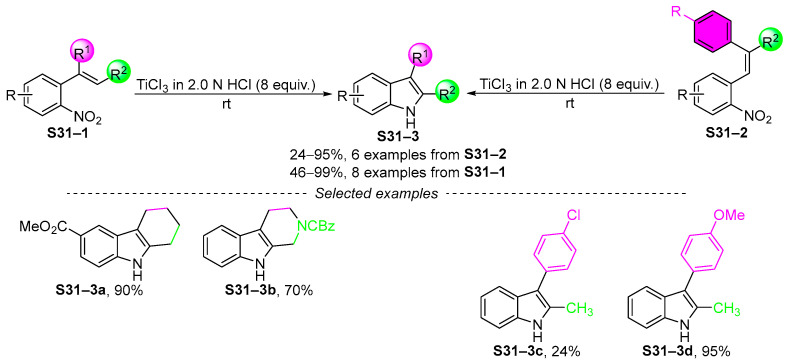
Efficient synthesis of substituted indoles from nitrobenzene derivatives containing a trisubstituted vinyl moiety.

Indole derivatives can also be synthesized from nitrobenzenes. A representative example is the preparation of indoles **S31–3** from benzene derivatives **S31–1** and **S31–2** (Scheme 31) [84]. The optimized reaction submodules are notable for their applicability to a wide range of trisubstituted alkenes—14 out of 27 examples. In the case of benzene derivatives **S31–1**, ester functionalities are well tolerated. In contrast, alkenes **S31–2** undergo cyclization to indole derivatives **S31–3** with concurrent migration of the alkyl group. According to the proposed mechanism, the nitro group is reduced in situ to either a nitroso or hydroxylamine intermediate, which subsequently undergoes cyclization to form indoles. The authors applied the optimized reaction conditions for the synthesis of rizatriptan; however, in that case, a disubstituted alkene was used as the starting material.

**Scheme 32:**
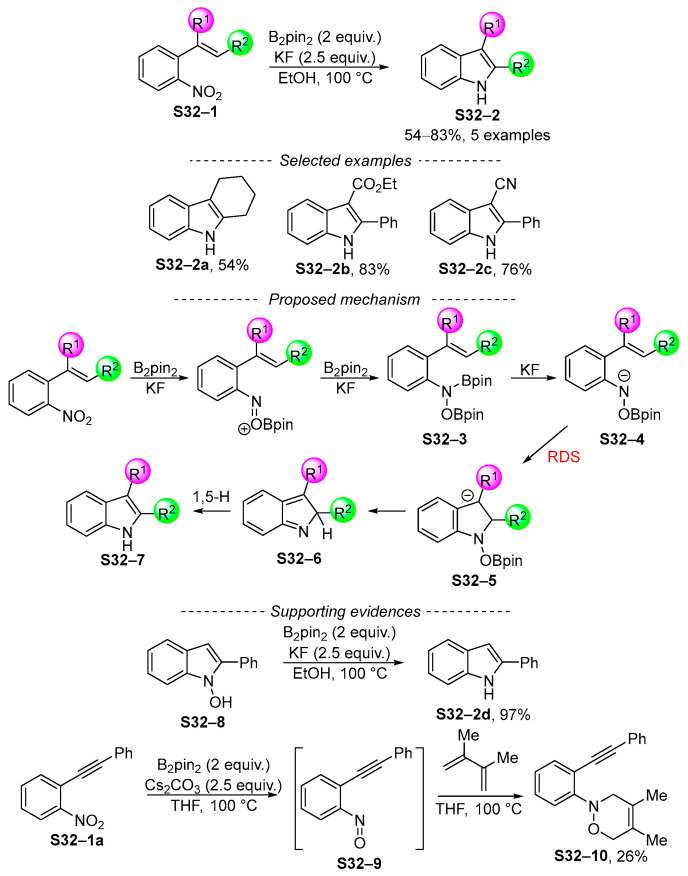
Diborane-mediated cyclization of nitrostyrenes.

Pinacolborane has also been employed in the cyclization of nitrobenzenes to indoles (Scheme 32) [85]. Optimal conditions involve the use of potassium fluoride in ethanol at 100 °C. Among the alkenes tested—primarily vicinal disubstituted alkenes—five trisubstituted variants were also examined. A detailed mechanistic investigation revealed that pinacolborane first reduces the nitro group to a nitrosobenzene intermediate, which is subsequently converted into an anionic species **S32–4**. Cyclization of this anionic intermediate is proposed to be the rate-determining step, as supported by Hammett analysis. This is consistent with the observation that the highest yields were obtained for alkenes bearing electron-withdrawing ester and nitrile substituents **S32–2b** and **S32–2c**. The final stage of the transformation involves a 1,5-hydrogen shift. Evidence for the formation of the nitroso intermediate was further supported by its successful interception in a [4+2] cycloaddition with 2,3-dimethylbuta-1,3-diene, yielding product **S32–10** in 26% isolated yield. In addition, the corresponding hydroxylamine intermediate **S32–8** was deoxygenated to the indole product **S32–2d** in quantitative yield.

**Scheme 33:**
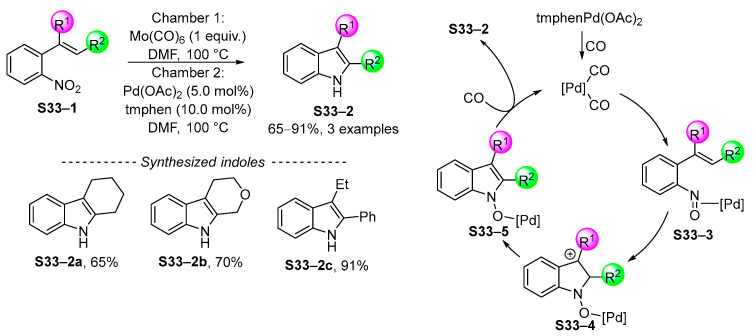
Palladium-catalyzed intramolecular cyclization of trisubstituted alkenes in the presence of molybdenum hexacarbonyl.

The reduction and subsequent cyclization of nitrobenzenes bearing a disubstituted vinyl group in the ortho position can also be catalyzed by a palladium complex (Scheme 33) [86]. In this methodology, molybdenum hexacarbonyl is employed as a source of carbon monoxide. According to the proposed mechanism, carbon monoxide serves a dual role: it reduces the Pd(II) species to a Pd(0)(CO)_2_ complex and also mediates the reduction of the intermediate **S33–5** to the final product **S33–2**. The substrate scope with respect to trisubstituted alkenes is narrow, with only 3 examples out of a total of 24 demonstrating successful conversion.

**Scheme 34:**
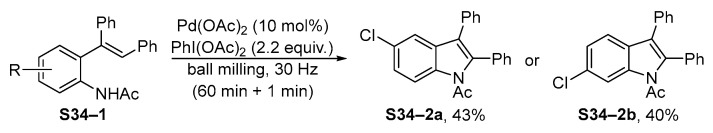
Mechanosynthesis of trisubstituted indoles.

A palladium-catalyzed intramolecular cyclization of trisubstituted alkenes **S34–1** has also been reported (Scheme 34) [87]. The scope of the reaction is limited to six examples, all featuring substitution on the aniline core. Notably, the use of ball milling is essential for achieving successful cyclization. Selected indole derivatives **S34–2a** and **S34–2b** demonstrate that the reaction conditions are compatible with halogen substituents. The starting trisubstituted alkenes were synthesized via rhodium-catalyzed hydroarylation of disubstituted alkynes under ball-milling conditions.

**Scheme 35:**
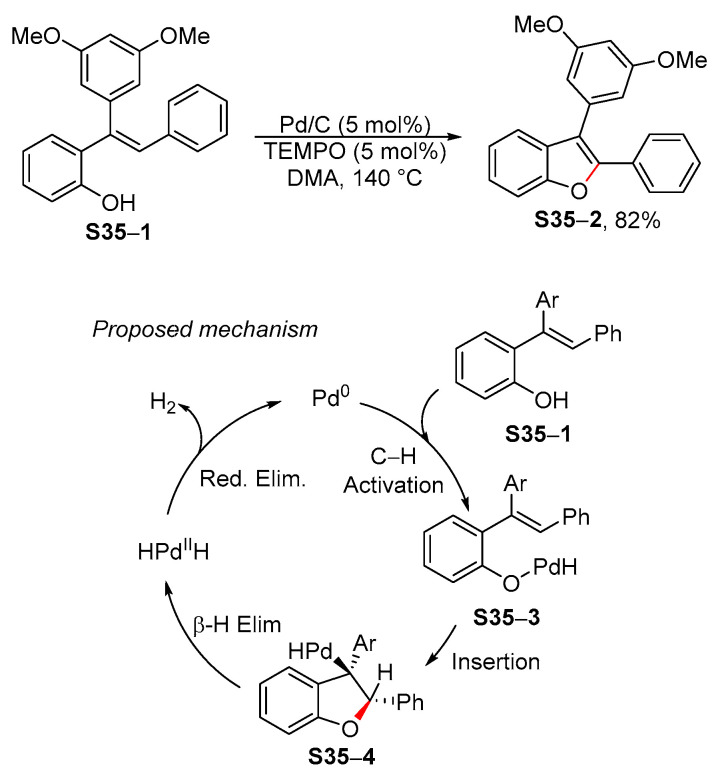
Palladium-catalyzed cyclization of phenols for the synthesis of benzofurans.

The methodology developed for the cyclization of aromatic amines was extended to the synthesis of benzofuran derivatives (Scheme 35) [88]. The published procedure involves a Pd(0)-catalyzed cyclization of phenols under straightforward reaction conditions. The authors proposed a mechanism that includes the activation of the phenolic O–H bond, followed by the syn-insertion of the double bond and subsequent β-H elimination. The catalytic species is regenerated via reductive elimination of hydrogen. However, the reaction suffers from a limited substrate scope. Of the 27 substrates tested, only 2 were trisubstituted alkenes. The cyclization of an unsymmetrically substituted double bond **S35–1** is illustrated in Scheme 35.

**Scheme 36:**
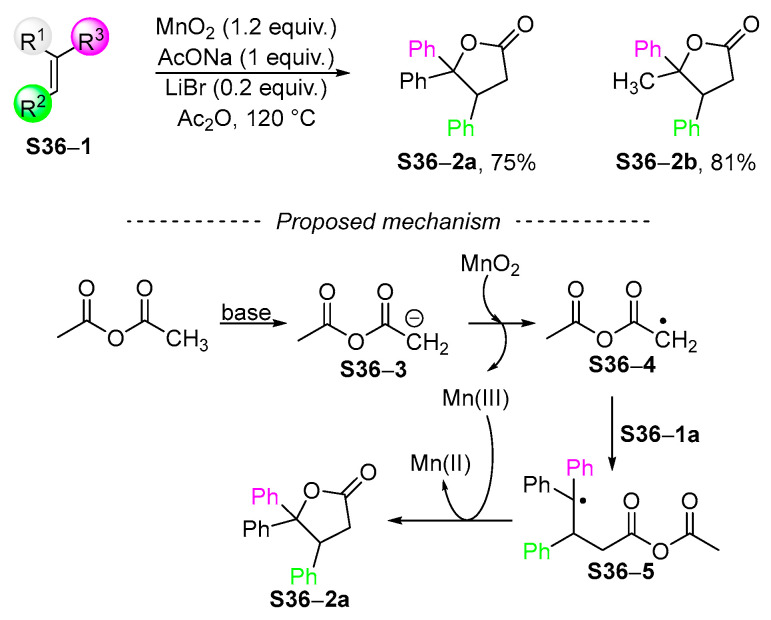
Cyclization of carboxylic acid derivatives mediated by manganese dioxide.

Intermolecular lactonization of alkenes with acetic anhydride was reported by Jiang and co-workers in 2015 (Scheme 36) [89]. According to the proposed mechanism, manganese dioxide serves as an electron source to generate the carbon-centered radical **S36–4** from acetic anhydride. This radical then adds to the alkene, forming the more stabilized intermediate **S36–5**. A Mn(III)-mediated radical–polar crossover followed by lactone ring closure affords the final products **S36–2a** and **S36–2b**. The authors also suggest that lithium bromide may assist in stabilizing the free radicals [90]. However, the scope of the reaction is largely limited to monosubstituted ethylenes. Only two trisubstituted alkenes out of the 29 tested derivatives were examined—one being the symmetrical triphenylethylene and the other an unsymmetrical derivative. No information is provided regarding the stereochemical purity of alkene **S36–1**.

**Scheme 37:**
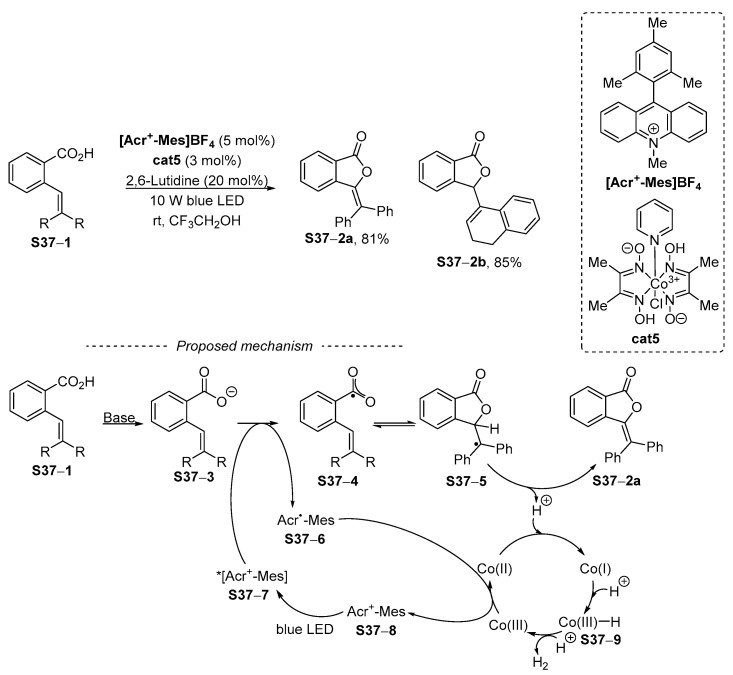
Visible-light-induced lactonization of trisubstituted alkenes. The asterisk (*) indicates a light-activated particle.

The lactonization of aromatic carboxylic acids was reported in 2018, employing cooperative catalysis by aridine and cobalt (Scheme 37) [91]. Although the reaction conditions were optimized primarily for benzene derivatives, they were also found applicable for the synthesis of lactones **S37–2a** and **S37–2b** from trisubstituted alkenes. Based on a series of experiments, a putative mechanism has been proposed and supported, involving the formation of the carboxylate ion **S37–3**, which is subsequently oxidized to the radical **S37–4** via a single-electron transfer (SET) process. The addition of the radical to the alkene affords the cyclic adduct **S37–5** that, presumably through a hydrogen atom transfer (HAT) step, is converted into the reaction product **S37–2a**. The yields of lactones **S37–2a** and **S37–2b** are comparable to those obtained for aromatic derivatives. Lactones with a comparable substrate scope can also be synthesized under electrochemical conditions [92].

**Scheme 38:**
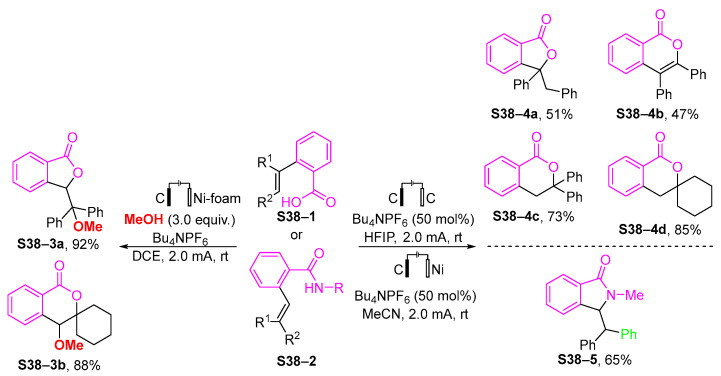
Electrochemical transformation of carboxylic acids and carboxamides into heterocyclic compounds.

An interesting transformation of carboxylic acids and carboxamides into heterocyclic products was reported by Park and co-workers (Scheme 38) [93]. In their study, carboxylic acids **S38–1** and their derivatives **S38–2** were subjected to electrochemical cyclization under varying conditions. Depending on the setup, carbon or nickel cathodes in HFIP or acetonitrile were employed for the synthesis of lactones **S38–4** and lactams **S38–5**. In contrast, lactonization carried out in the presence of a nucleophile (MeOH) resulted in the formation of ether products **S38–3a** and **S38–3b**. However, it should be noted that the scope of the reaction with respect to trisubstituted alkenes is very limited and involves the formation of 7 compounds out of a total of 59 products prepared. The proposed mechanism involves a radical process and was supported by an electrochemical study.

**Scheme 39:**
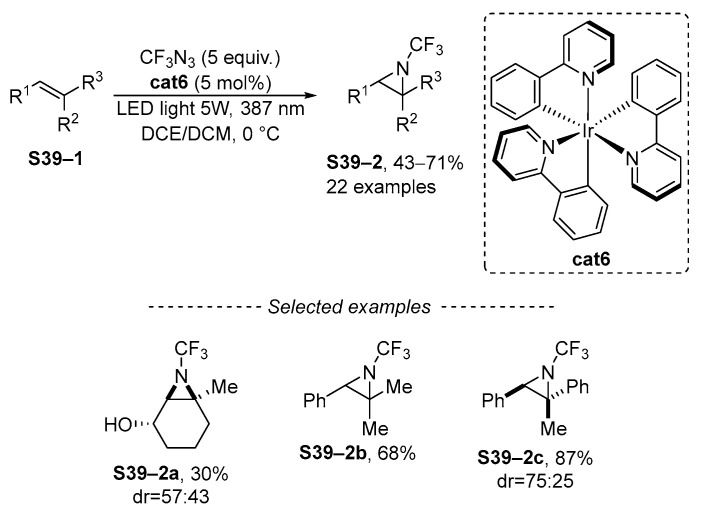
Iridium-catalyzed, blue light-mediated trifluoroamination of trisubstituted alkenes.

In 2024, Beier and co-workers reported the photochemical generation of a triplet trifluoromethyl nitrene, which was subsequently employed in the aziridination of trisubstituted alkenes (Scheme 39) [94]. The reaction conditions were evaluated across a broad range of alkene substitution patterns, including vicinally and geminally disubstituted, mono-, tri-, and tetrasubstituted alkenes. The substrate scope included 30 alkenes in total, among which 6 were trisubstituted derivatives. The resulting aziridines were obtained in good yields. However, in the case of stereoisomeric trisubstituted alkenes, the formation of diastereomeric mixtures **S39–2a** and **S39–2c** was observed. The authors proposed that the trifluoromethyl nitrene is generated in situ and directly added to the alkene double bond. This mechanistic hypothesis was supported by electron paramagnetic resonance (EPR) spectroscopy and corroborated by quantum chemical calculations.

**Scheme 40:**
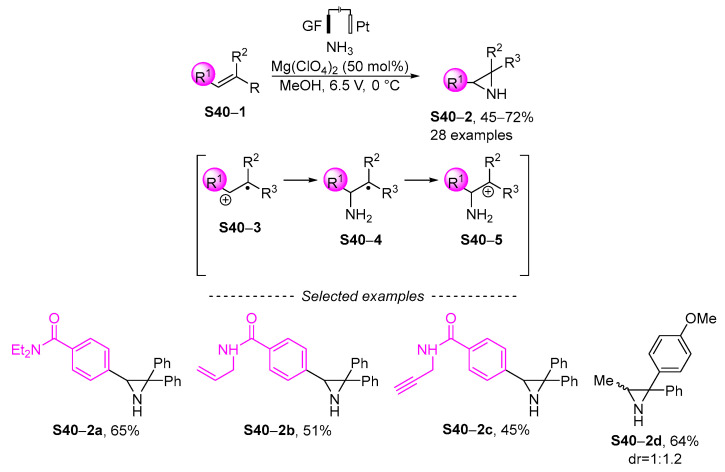
Electrochemical aziridination of trisubstituted alkenes.

Ammonia addition is a widely utilized transformation in organic synthesis, most commonly leading to aziridine derivatives—hence the term aziridination. In a series of studies, Liang, together with Cheng [95] and Cheng [96] reported electrochemical aziridination reactions involving trisubstituted alkenes. Their initial publication [96] in 2018 described the electrochemical aziridination of trisubstituted alkenes using primary amines in the presence of 2,6-lutidine and lithium chlorate in acetonitrile. In a subsequent study, the methodology was expanded to include the use of ammonia as the nitrogen source, enabling the aziridination of both tri- and tetrasubstituted alkenes (Scheme 40) [95]. The optimized reaction conditions involve magnesium chlorate as the supporting electrolyte, a graphite anode, and a platinum cathode. A notable feature of the method is its broad substrate scope, covering 28 trisubstituted alkenes. The reaction tolerates a variety of functional groups, including esters, amides, alkenes, and alkynes, as demonstrated by a series of representative aziridine products: **S40–2a**, **S40–2b**, **S40–2c**, and **S40–2d**. For unsymmetrically substituted alkenes, diastereomeric mixtures of aziridines were observed. The proposed mechanism, supported by quantum chemical calculations, involves initial anodic oxidation of the alkene to generate a radical cation **S40–3**, which undergoes nucleophilic attack by ammonia to form a carbon-centered radical **S40–4**. Further oxidation of this intermediate leads to a carbocation that cyclizes to form the final aziridine product, **S40–2**.

**Scheme 41:**
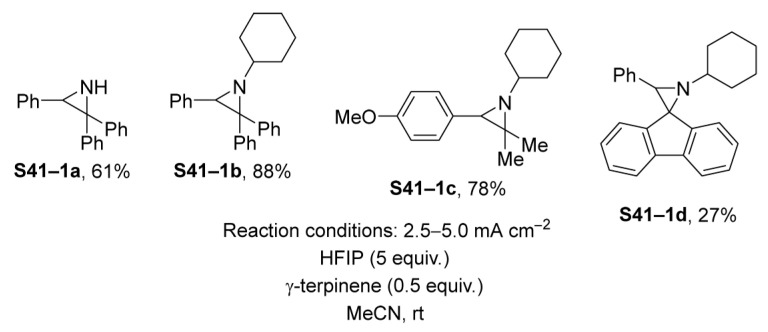
Electrochemical aziridination of trisubstituted alkenes performed in a continuous-flow electrochemical reactor.

An interesting advancement in the field of electrochemical aziridination is its implementation under continuous-flow electrochemical conditions (Scheme 41) [97]. This setup enables significantly shorter reaction times compared to conventional batch processes. Although the reaction was primarily optimized for disubstituted alkenes, several trisubstituted aziridines were also successfully synthesized, demonstrating the method’s broader applicability. Among the 28 alkenes tested, 4 trisubstituted substrates yielded the target aziridines **S41–1a**, **S41–1b**, **S41–1c**, and **S41–1d** efficiently.

**Scheme 42:**
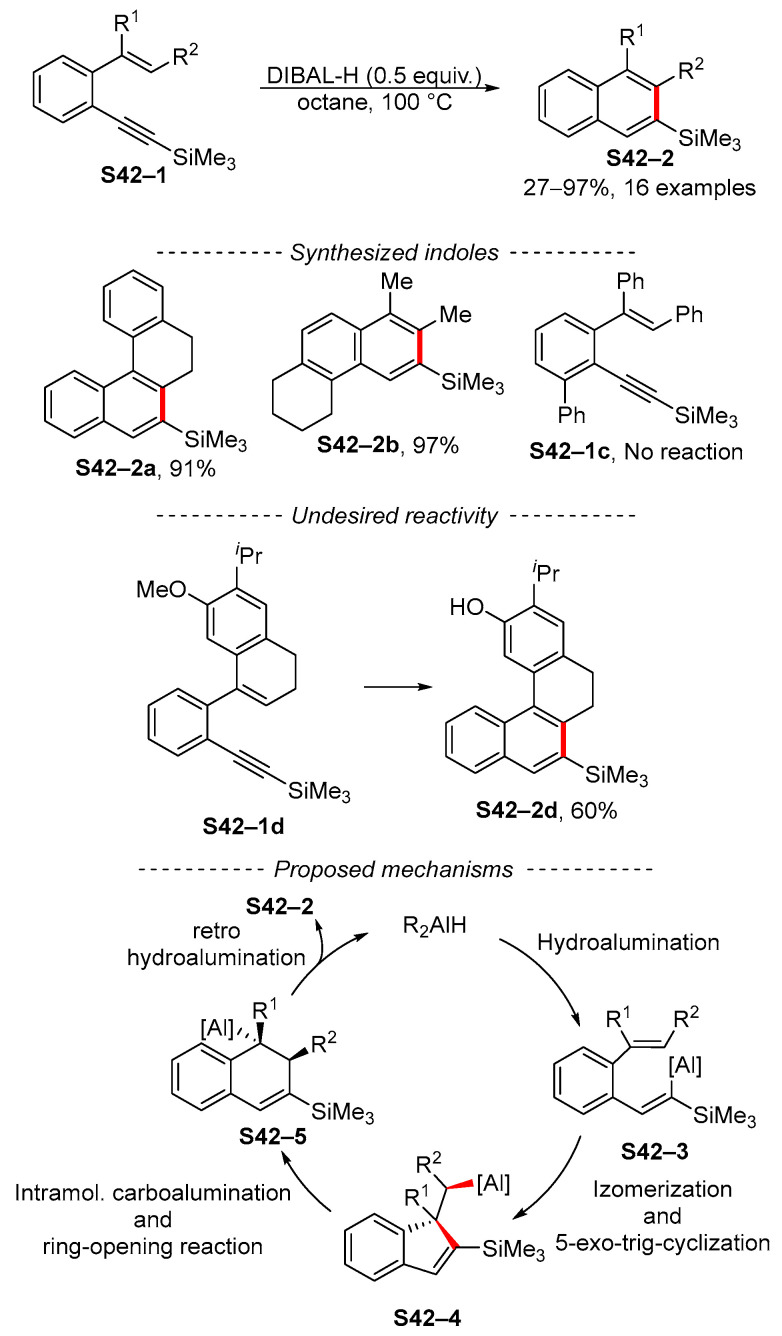
DIBAL-H-promoted cyclization of trisubstituted alkenes.

An interesting cyclization of enynes to naphthalene derivatives, notable for the number of trisubstituted alkenes involved, was reported by Miura (Scheme 42) [98]. The reaction featured a simple experimental setup, using DIBAL-H at 100 °C. The reported conditions were sensitive to substitution on the benzene ring, and ortho-substituted substrates were not tolerated, as demonstrated by enyne **S42**–**1c**, which remained unreactive under the optimized conditions. Due to the harsh reaction conditions, functional group tolerance was minimal. This was illustrated by enyne **S42–1d**, where the demethylation of the methoxy group was observed. According to the authors, the formation of the product involves both the hydroalumination and carboalumination of the alkyne and alkene moieties.

**Scheme 43:**
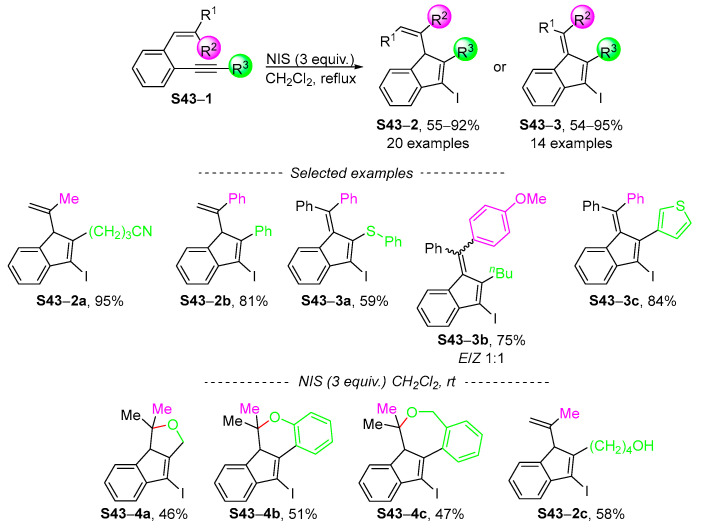
Iodocyclization of trisubstituted alkenes en route to indene derivatives.

By selecting appropriately structured starting materials, indene derivatives can be accessed through the cyclization of trisubstituted alkenes. A typical example is the iodocyclization of enynes, which proceeds under experimentally simple conditions—by treating the substrates with *N*-iodosuccinimide (NIS) in dichloromethane (Scheme 43) [99]. This reaction typically involves trisubstituted alkenes as the exclusive substrates. The structure of the resulting products strongly depends on the substitution pattern of the starting materials. In the case of aliphatic or mixed aliphatic–aromatic substituents, indene derivatives **S43-2** are typically formed. For alkenes bearing geminal diaryl substituents, the reaction instead affords conjugated dienes **S43-3**. A variety of substituted enynes **S43-1** were tested. Although halogens and alkoxy groups are tolerated, the general tolerance toward functional groups is limited, with nitrile groups being the most reliably tolerated. The substitution pattern on both the double and triple bonds critically influences the outcome. The cyclization of substrates containing a hydroxy group in the side chain results in the formation of five-, six-, or seven-membered rings **S43-4a**, **S43-4b**, and **S43-4c**. However, formation of eight-membered rings has not been observed. Notably, cyclization in these cases proceeds under milder reaction conditions. Finally, the authors demonstrated that the iodocyclized products can be further functionalized through iodine–lithium exchange followed by palladium-catalyzed cross-coupling reactions.

**Scheme 44:**
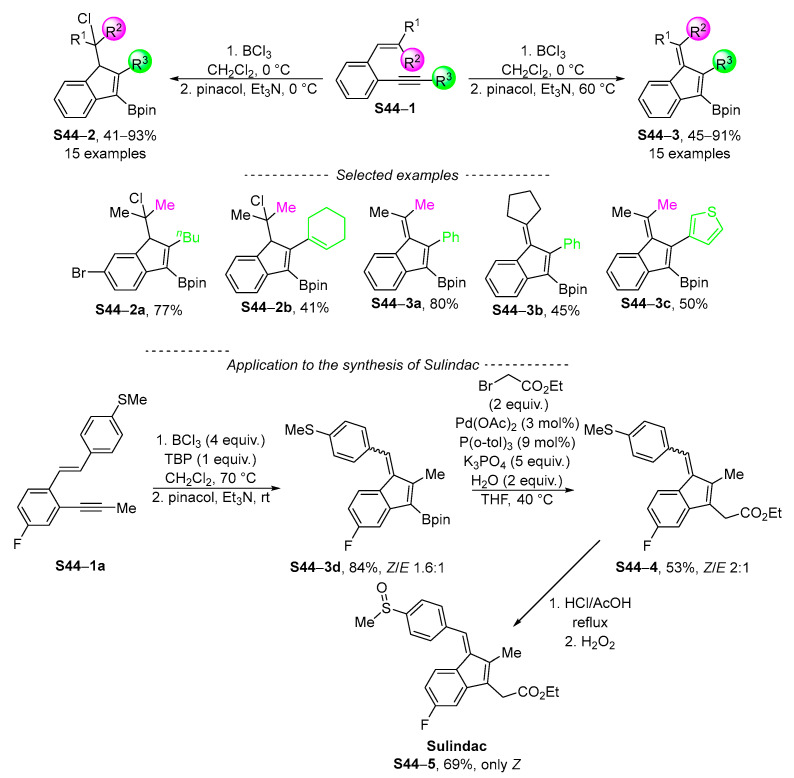
Synthesis of borylated indene derivatives via BCl_3_-mediated cyclization of trisubstituted alkenes.

The previously described reaction conditions for the iodocyclization of trisubstituted alkenes were applied to the borylative cyclization of enynes (Scheme 44) [100]. In this process, the starting enynes undergo cyclization in the presence of boron trichloride (BCl_3_). The nature of the final products is strongly dependent on the reaction conditions. When the reaction with pinacol is performed at 0 °C, indene derivatives **S44–2** are obtained. In contrast, carrying out the same reaction at 60 °C results in the formation of conjugated diene **S44–3**. These observations suggest that base-induced dehydrohalogenation of the intermediate chloro derivative **S44–2** is responsible for the formation of diene **S44–3**. The optimized transformation tolerates a broad range of trisubstituted alkenes, although the functional group tolerance remains limited. Notably, borylative cyclization has also been employed in the synthesis of sulindac, starting from a disubstituted alkene **S44–1a**. In this case, the cyclization requires harsher conditions and results in a mixture of *Z* and *E* stereoisomers. A subsequent cross-coupling with ethyl bromoacetate likewise produces a stereoisomeric mixture. However, during hydrolysis of the ester and oxidation to the sulfoxide **S44–5**, only the *Z* isomer of sulindac was obtained, indicating a stereoselective outcome in the final steps.

**Scheme 45:**
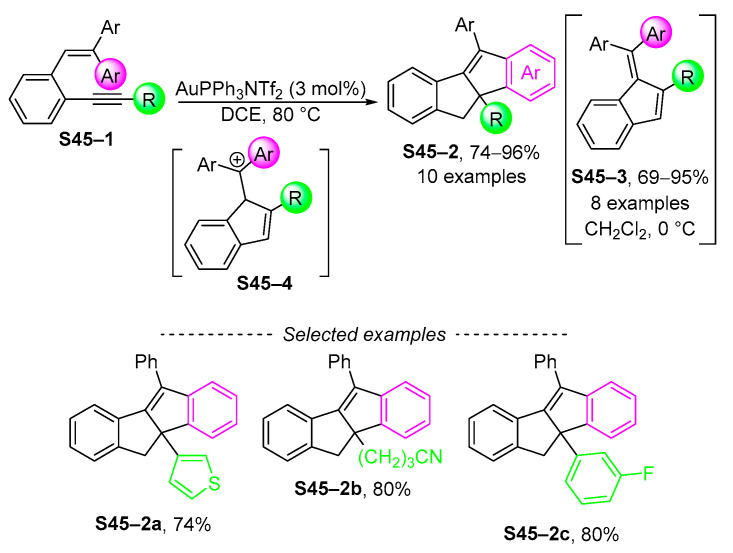
Gold-catalyzed cyclization of enynes.

The similar cyclization of enynes to indene derivatives can also be efficiently catalyzed by a gold complex under mild conditions (CH_2_Cl_2_, room temperature), affording indene derivatives **S45–3**. Upon changing the solvent and increasing the reaction temperature to 80 °C, a double cyclization takes place, leading to the formation of a tetracyclic product **S45–2** (Scheme 45) [101]. The substrate scope includes ten trisubstituted alkenes, although the range of tolerated functional groups is narrow, being limited to nitrile substituents. The authors propose that the formation of products **S45–2** and **S45–3** proceeds via the carbocationic intermediate **S45–4**, which plays a key role in the reaction mechanism.

**Scheme 46:**
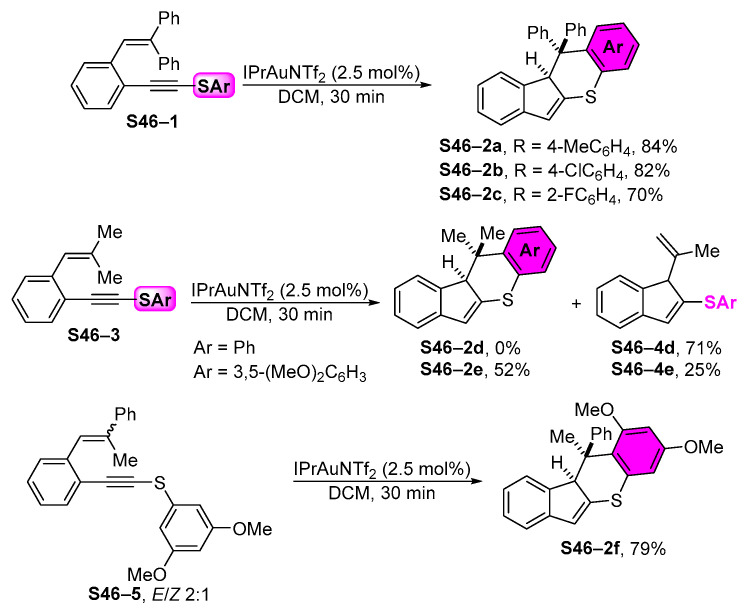
Gold-catalyzed cycloisomerization of trisubstituted alkenes to dihydroindeno[2,1-b]thiochromenes.

Dihydro[2,1-b]thiochromene derivatives can be synthesized from alkynyl(aryl)sulfides under gold catalysis. Optimal conditions involve the use of IPrAuNTf_2_ as a catalyst in dichloromethane at room temperature. Under these conditions, disubstituted alkenes are converted to thiochromene derivatives within 30 min. The reaction was also successfully applied to a trisubstituted alkene including six trisubstituted alkenes out of the 30 tested (Scheme 46) [102]. The reaction outcome strongly depends on the substitution pattern of both the alkene and the alkyne. Triaryl ethylenes **S46–1** furnished the tetracyclic products **S46–2a**, **S46–2b**, and **S46–2c** in high isolated yields. An alkene bearing a single methyl group at the double bond afforded the corresponding tetracyclic product **S46–2f** with high diastereoselectivity. In contrast, ethylenes bearing geminal dimethyl groups gave either the indene derivative **S46–4d** or a mixture of the indene **S46–4e** and the tetracyclic product **S46–2e**, depending on the substitution pattern of the alkyne unit. The formation of bicyclic and tetracyclic products is proposed to proceed via a common cyclopropyl gold carbene intermediate, which undergoes divergent transformations leading to compounds **S46–2** and **S46–4**. However, the effect of the alkene substitution pattern on product distribution was not discussed in detail.

**Scheme 47:**
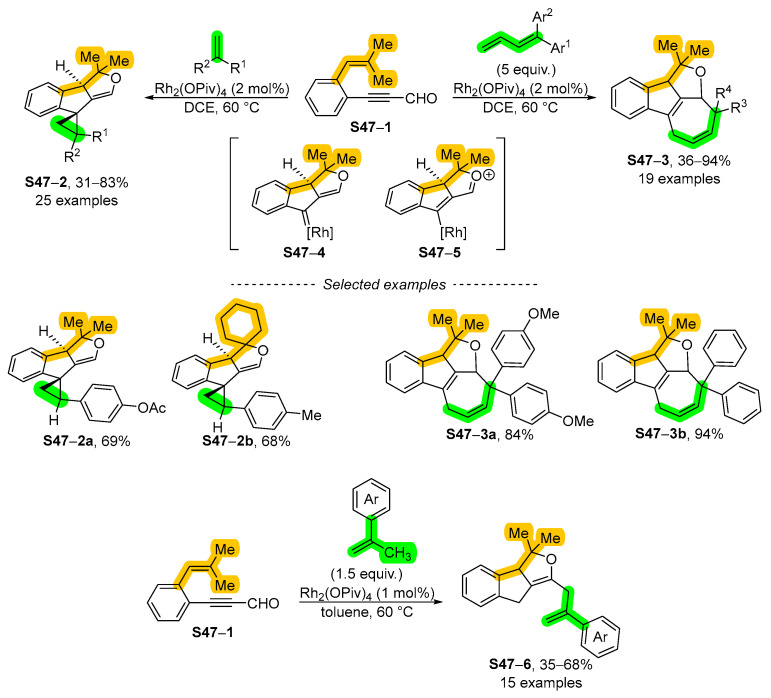
Rhodium-catalyzed [1+2] and [3+4] cycloaddition of enynes.

A similar cyclization of enynes can also be catalyzed by rhodium complexes. Depending on the structure of the alkene, the cyclization of enyne **S36–1** leads to the tricyclic product **S47–2** (Scheme 47) [103]. In contrast, the use of a conjugated diene allows access to the tetracyclic product **S47–3**. An illustrative example is the synthesis of compounds **S47–2a**, **S47–2b**, **S47–3a**, and **S47–3b**, obtained from the corresponding trisubstituted alkenes through reactions 1,1-disubstituted alkenes and substituted buta-1,3-diene. By modifying the reaction conditions—specifically, by using toluene as the solvent—alternative cyclic **S47–6** can be formed. The authors also explored an enantioselective version of the transformation. A satisfactory enantiomeric ratio (er ≥79:21) was achieved using 2 mol% of the Rh_2_(S-NTTL)_4_ complex in dichloromethane. A plausible mechanism was proposed, involving key cyclic intermediates **S47–4** and **S47–5**, which are believed to react with alkenes to afford the final products.

**Scheme 48:**
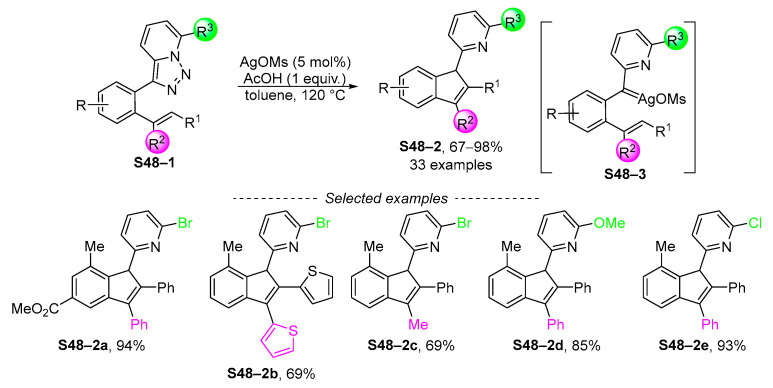
Silver(I)-mediated activation of triazoles en route to indene derivatives.

A mechanistically distinct synthesis of indene derivatives is based on triazole precursors **S48–1** (Scheme 48) [104]. The reaction, which predominantly employs trisubstituted alkenes—35 out of the 37 tested—requires a silver catalyst and acetic acid. The authors proposed a mechanism for the formation of indene derivatives **S48–2** involving denitrogenation to generate a silver carbene intermediate, **S48–3**, which subsequently reacts with the trisubstituted double bond. The proposed pathway is supported by quantum chemical calculations and deuterium-labeling experiments.

**Scheme 49:**
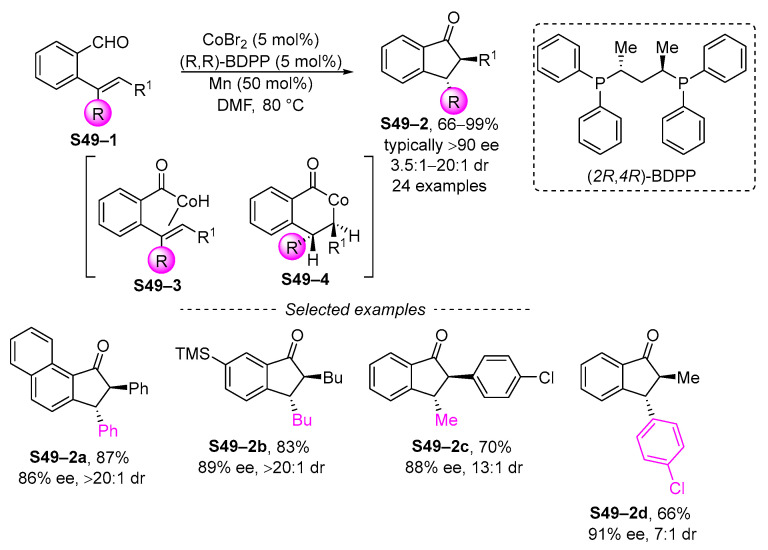
Enantioselective cobalt-catalyzed cyclization of trisubstituted alkenes.

In 2017, the enantioselective cyclization of enals was reported (Scheme 49) [105]. The reaction is notable for the exclusive use of trisubstituted alkenes, comprising 24 examples—mostly as mixtures of *E*/*Z* isomers with *E*/*Z* ratios ranging from 1:2 to 1:20. According to the authors, the key steps in the formation of the cyclic products **S49–2** involve the oxidative addition of cobalt to the C–H bond, resulting in the complex **S49–3**. This step is followed by hydrometallation to give the complex **S49–4**. The proposed mechanism is supported by deuterium-labeling experiments and literature precedents.

**Scheme 50:**
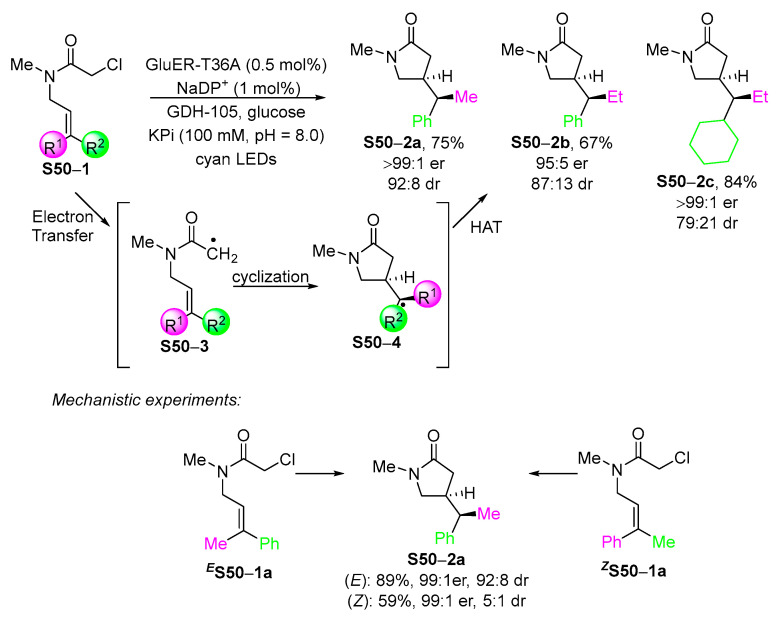
Photocatalytic diastereoselective cyclization of trisubstituted alkenes.

Trisubstituted alkenes were also employed in the enzyme-catalyzed radical cyclization (Scheme 50) [106]. The starting alkene **S50–1** undergoes cyclization to form lactams with high diastereoselectivity and enantioselectivity. Optimized reaction conditions were investigated for 16 alkenes, of which only three were trisubstituted. Cyclization of these trisubstituted alkenes afforded the products **S50–2a**, **S50–2b**, and **S50–2c**. The reaction is catalyzed by the flavin-dependent ene-reductase GluER-T36A. As part of this experimental study, the formation of radical **S50–3** was proposed to occur via electron transfer within a donor–acceptor complex formed between the alkene and the reduced flavin cofactor. The intermediate radical **S50–3** undergoes intramolecular cyclization, followed by hydrogen atom abstraction (HAT) to yield the final products. It has been proposed that, upon formation of radical **S50–3**, the enzyme facilitates hydrogen atom transfer selectively from a single rotamer of the prochiral intermediate, with the HAT rate being comparable to the rate of C–C bond rotation. The enantioselectivity of the reaction is independent of the configuration of the trisubstituted double bond, as demonstrated by mechanistic experiments. The same diastereomer is preferentially formed from both *E* and *Z* isomers.

**Scheme 51:**
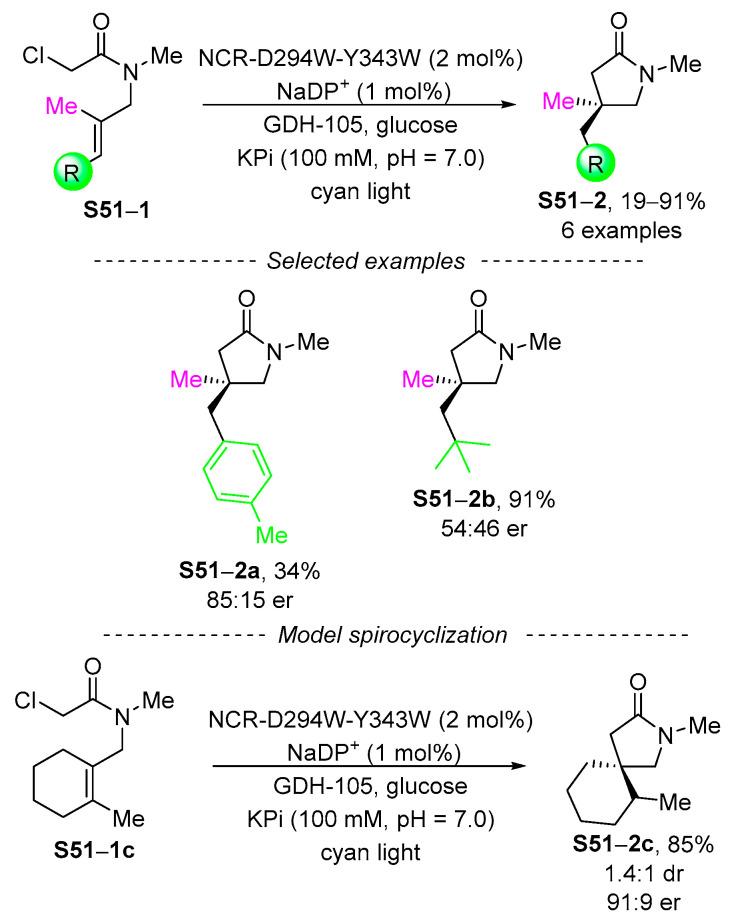
Photoinduced enantioselective cyclization of trisubstituted alkenes.

Building on previous results [106], Hyster and colleagues extended the methodology of biocatalytic asymmetric cyclization of chloroacetamides to the synthesis of cyclic lactams **S51–2** (Scheme 51) [107]. The developed reaction conditions were applied to the cyclization of seven trisubstituted alkenes, including one cyclic alkene, which afforded the spirocyclic product **S51–2c**. The cyclization proceeded with lower enantioselectivity, ranging from 54:46 to 85:15, as illustrated by examples **S51–2a** and **S51–2b**.

**Scheme 52:**
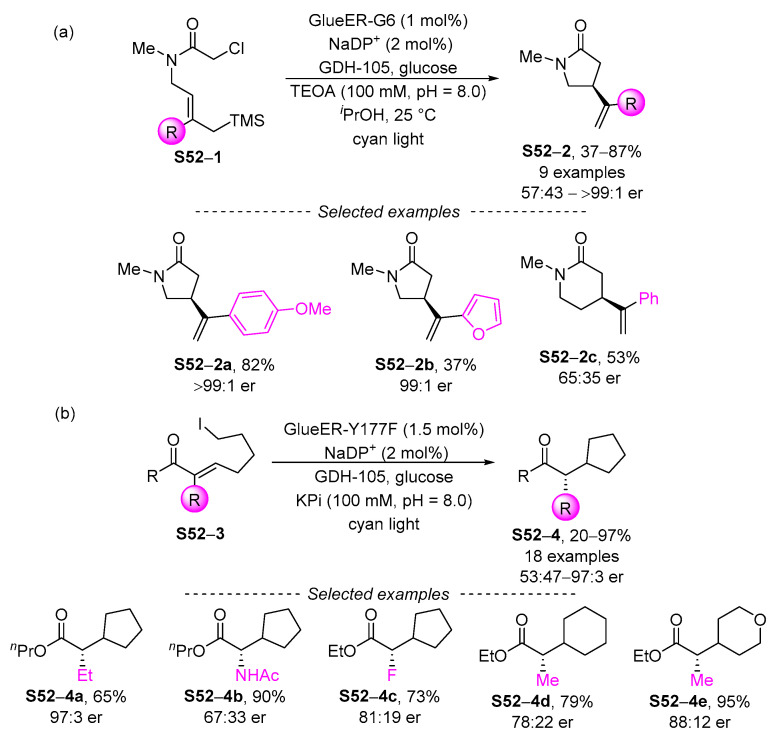
(**a**) Enzyme-catalyzed cyclization of chloroacetamides and (**b**) α,β-unsaturated esters.

An interesting extension of enzymatically catalyzed radical cyclizations of trisubstituted alkenes is the synthesis of cyclic lactams involving β-scission of the TMS group (Scheme 52a) [108]. The reaction was primarily explored for the preparation of five-membered lactams, but it can also be applied to the synthesis of a six-membered derivative **S52–2c**, albeit with limited stereoselectivity. Conversely, intramolecular cyclization of trisubstituted α,β-unsaturated alkenes affords cyclopentane derivatives **S52–4** (Scheme 52b) [109]. In certain cases, this methodology can also be extended to the formation of cyclohexane derivatives.

**Scheme 53:**
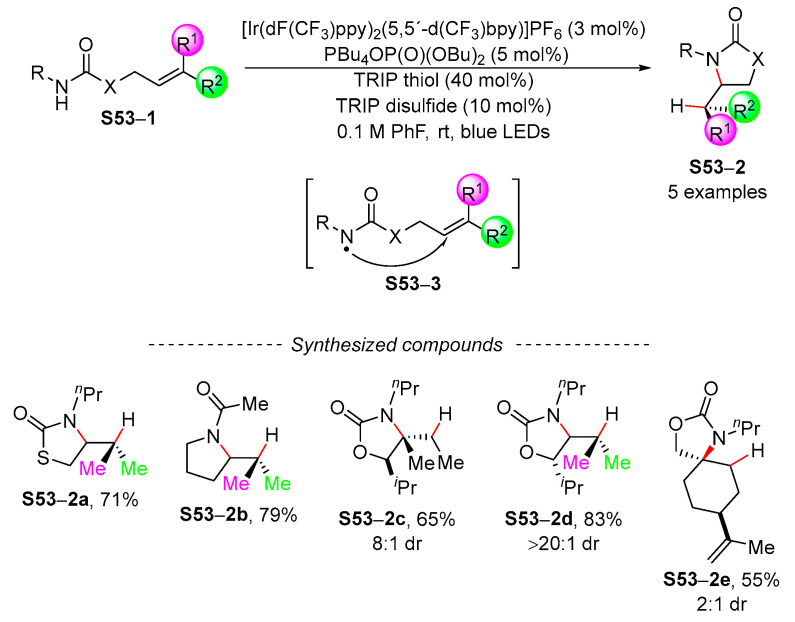
Intramolecular hydroamination of trisubstituted alkenes.

A logical extension of the hydroamination of trisubstituted alkenes is represented by its intramolecular variant (Scheme 53) [110]. Reaction conditions enabled the generation of an amidyl radical **S53–3**, which subsequently underwent intramolecular addition to the C=C double bond, affording carbamates and lactams, respectively. The optimized conditions were applied to a set of 34 alkenes, of which only 5 were trisubstituted.

**Scheme 54:**
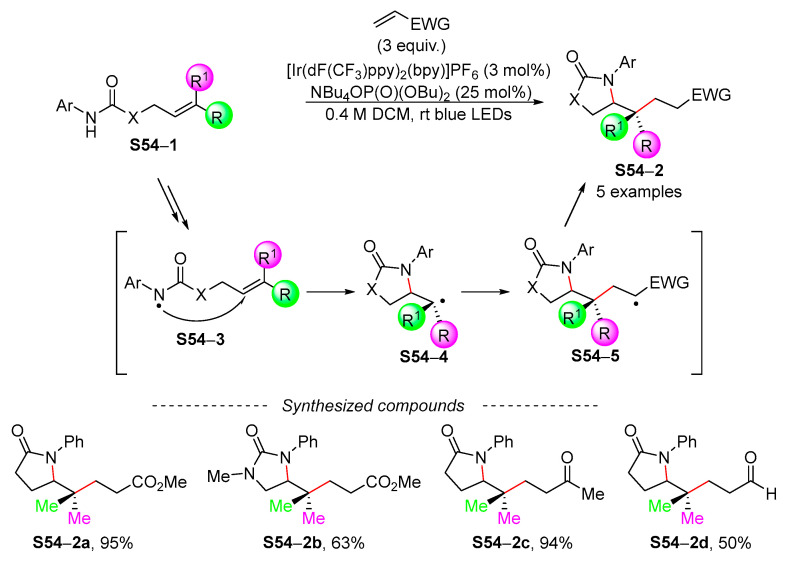
Catalytic hydroamination of trisubstituted alkenes followed by radical addition to the C=C double bond.

An interesting extension of the intramolecular hydroamination of trisubstituted alkenes involves its combination with intermolecular addition to terminal alkenes (Scheme 54) [111]. In this transformation, the amidyl radical **S54–3** undergoes intramolecular addition to a triple bond, generating the alkyl radical **S54–4**, which subsequently reacts with an electron-deficient alkene to furnish the target compounds **S54–2**. The reaction demonstrates a broad substrate scope with respect to trisubstituted alkenes—20 out of 24 tested substrates fall into this category. Another noteworthy feature is the tolerance of highly reactive functional groups, including keto and aldehyde moieties **S54–2c** and **S54–2d**, which are often incompatible under radical conditions.

**Scheme 55:**
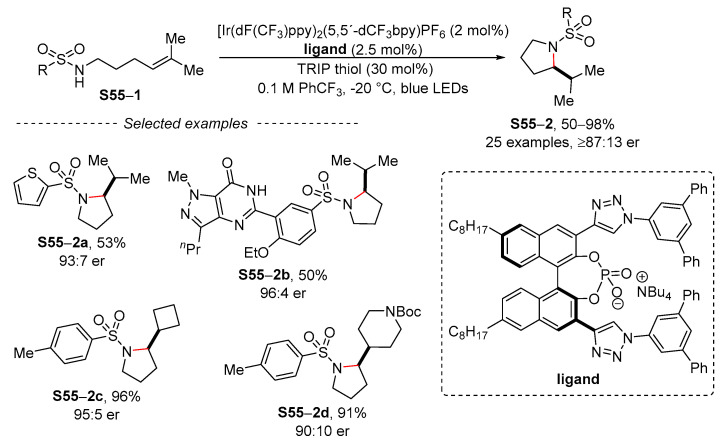
Enantioselective cyclization of sulfonamides.

Enantioselective intramolecular hydroamination of trisubstituted alkenes was reported in 2020 (Scheme 55) [112]. High enantioselectivity was achieved through the cyclization of sulfonamides in the presence of a sophisticated chiral ligand under low-temperature conditions. Elevation of the reaction temperature to ambient levels led to a significant drop in enantioselectivity. This study employed 21 trisubstituted alkenes bearing a dimethylvinyl group and various sulfonamide moieties. Additionally, cyclic sulfonamides bearing cyclobutyl **S55–2c** and piperidyl **S55–2d** substituents were also synthesized. The high enantioselectivity was later attributed to noncovalent association between the radical intermediate and the chiral environment provided by the phosphoric acid catalyst [113].

**Scheme 56:**
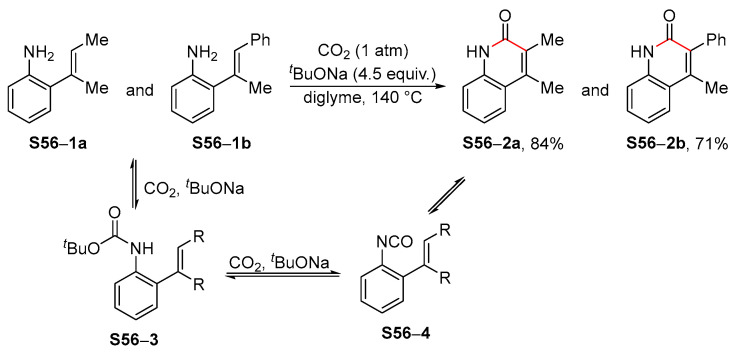
Transition-metal-free cyclization of trisubstituted alkenes.

Chemical modifications of trisubstituted alkenes also include carboxylation reactions, in which various trisubstituted alkenes react with carbon dioxide under appropriate reaction conditions. Transition metal-catalyzed carboxylations include, for example, the lactamization to 2-quinolinones (Scheme 56) [114]. Although the optimized conditions were evaluated on a broad range of alkenes, mostly disubstituted alkenes, only two trisubstituted alkenes were tested. Inductively coupled plasma atomic emission spectroscopy (ICP-AES) analysis revealed that the reaction proceeds in the absence of a transition metal, and the authors proposed the formation of two key intermediates, **S56–3** and **S56–4**, as essential for the formation of 2-quinolinones.

**Scheme 57:**
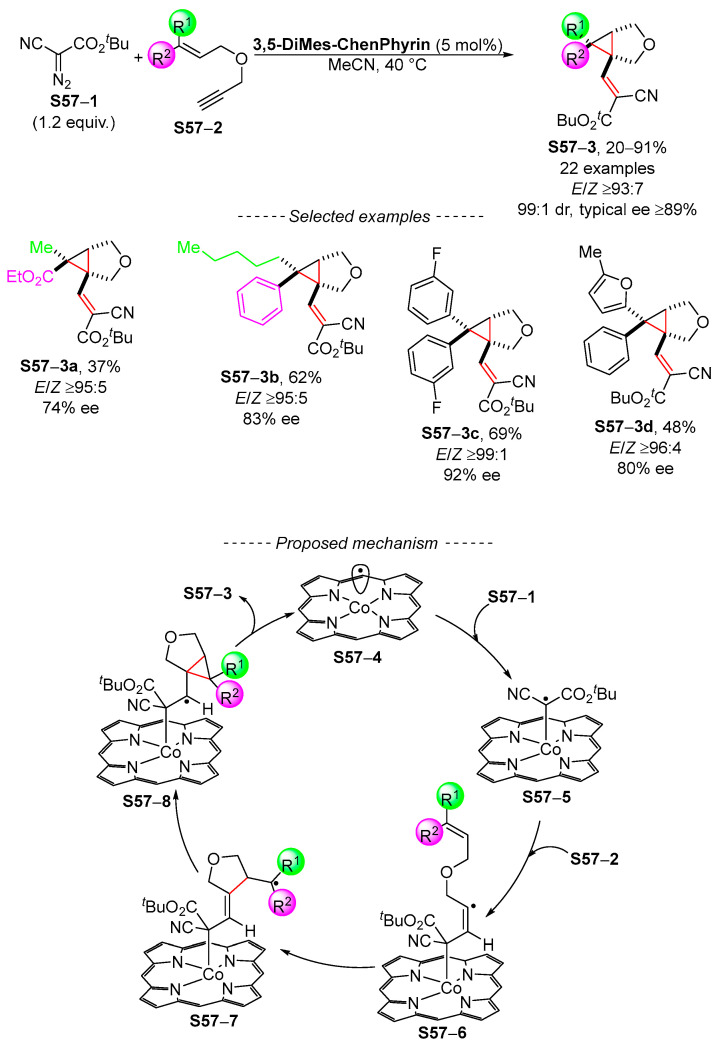
Radical cyclization of trisubstituted alkenes via metalloradical catalysis.

Recently, Zhang’s research group reported a series of radical cyclizations of trisubstituted alkenes enabled by metalloradical catalysis. This strategy allowed for the synthesis of various bicyclic aziridines [115,116] and cyclopropanes [117,118]. While the reactions were thoroughly investigated for mono- and disubstituted alkenes, their applicability to trisubstituted alkenes proved limited, with only a single example reported. A notable exception within this body of work is the intramolecular cyclopropanation of enynes **S57–2** with the diazo compound **S57–1,** catalyzed by a 3,5-diMes-ChenPhyrin cobalt complex (Scheme 57) [119]. The use of a chiral cobalt catalyst ensures high enantioselectivity of the cyclization. The proposed mechanism involves the initial reaction of the diazo compound to generate a cobalt-stabilized radical **S57–5**, which adds to the alkyne moiety to form a vinyl radical intermediate. This species subsequently undergoes a 5-exo-trig cyclization, followed by a second ring closure, to afford a strained intermediate complex. A β-scission of this complex yields the cyclopropane product alongside regeneration of the catalyst. The mechanism was supported by experimental data, quantum chemical calculations, and EPR spectroscopy. The reaction proceeds with excellent stereoselectivity. Moreover, the authors demonstrated that the C=C double bond in the resulting molecule can be further transformed via oxidative cleavage, epoxidation, or reaction with Grignard reagents.

## 3. Conclusions

In this review, we have summarized the use of trisubstituted alkenes in organic synthesis. The discussed transformations include additions of carbon-, nitrogen-, and oxygen-based nucleophiles, as well as carboxylation reactions. The second section focuses on the oxidative cleavage of trisubstituted alkenes, while the final part covers their cyclization, particularly into indole and indene derivatives, along with other five- and three-membered heterocycles.

These transformations can be evaluated in terms of the diversity of accessible structural motifs and their tolerance to functional groups. Another important aspect is the number of trisubstituted alkenes tested under the reported conditions. From the perspective of structural diversity and functional group compatibility, the described methods allow for the preparation of a broad spectrum of acyclic and cyclic products, including various heterocycles. However, in terms of functional group tolerance, the methodologies remain limited and frequently exclude sensitive moieties such as aldehydes and ketones.

A notable limitation of many of these transformations is the relatively small number of trisubstituted alkenes that have been explored. In numerous studies, the reaction conditions were optimized primarily using disubstituted alkenes, likely due to their greater availability. When trisubstituted alkenes were included, only a limited number of examples were typically tested. An exception to this trend is seen in cyclization reactions, which have often been studied using extensive libraries of trisubstituted alkenes. This broader applicability is largely due to the tolerance of both (*E*)- and (*Z*)-isomers in these reactions, allowing for the use of stereoisomeric mixtures and simplifying practical application.

These observations lead to two key conclusions: first, a major limitation of current methodologies involving trisubstituted alkenes lies in their insufficient tolerance—or more precisely, testing—of substrates bearing highly reactive functional groups. Second, the limited availability of stereochemically pure trisubstituted alkenes represents a further bottleneck for broader application.

## Figures and Tables

**Table 1:** Overview of the oxidative cleavage of C=C bonds in trisubstituted alkenes.

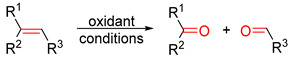				
Entry	Conditions	Intermediates	Alkenes	Selected Products
1	O_3_ EtOAc:H_2_O	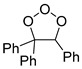	1/10	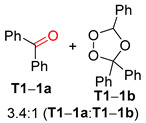
2	H_2_O_2_ (1.8 equiv.) (c-C_6_H_11_Se)_2_ (4 mol%) Fe(NO_3_)_2_ (4 mol%) Acetone, 80 °C, O_2_	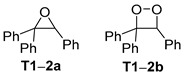	6/27	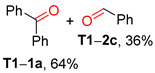
3	H_2_O_2_ (5.0 equiv.) (RSe)_2_ (5 mol%) EtOH, 80–120 °C	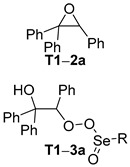	13/28	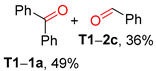
4	H_2_O_2_ (3.0 equiv.) **Cat5** (10.0 mol%) MeCN, rt	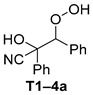	3/10	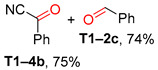
5	**Cat6** (1.0 mol%) TBAI (10 mol%) NaIO_4_ (2.0 equiv.) H_2_O, rt	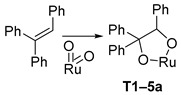	1/15	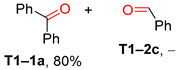
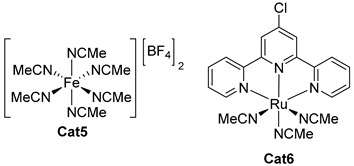				

**Table 2:** Overview of the oxidative cleavage of C=C bonds in trisubstituted alkenes.

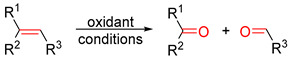				
Entry	Conditions	Intermediate	Alkenes	Selected Products
1	O_2_ PEGDME 110 °C	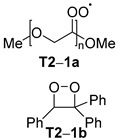	2/34	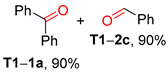
2	PhSO_2_Na, O_2_ 30 W purple LED, DCE	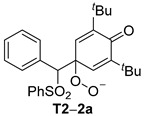	1/40	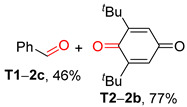
3	4-NO_2_(C_6_H_4_)CN (1.5 equiv.) 390 nm MeCN, 23 °C	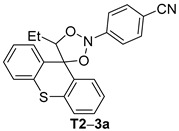	11/39	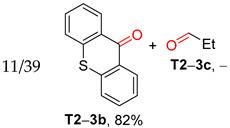
4	Mn(dtbpy)_2_(OTf)_2_ (2 mol%) MeOH/THF blue light (9 W, 470 nm), 20 °C, O_2_	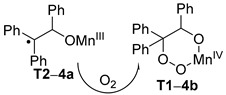	3/80	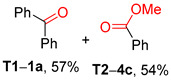

**Table 3:** Cyclization of trisubstituted alkenes en route to indole derivatives.

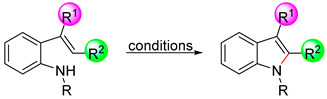				
Entry	Conditions	Intermediate	Alkenes	Selected Products
1	NIS (2.0 equiv.) DCM, rt	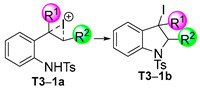	4/41	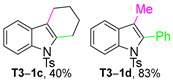
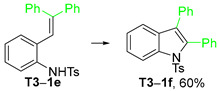				
2	Ag_2_CO_3_ (1.3 equiv.) DMF, 150 °C	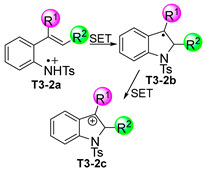	8/50	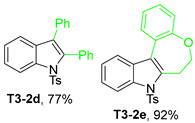
3	Benzoquinone (1.0 equiv.) TsOH•H_2_O (20 mol%) 1,4-dioxane, 80 °C	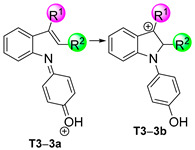	12/23	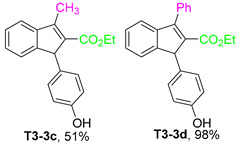

## Data Availability

No new data were created or analyzed in this study. Data sharing is not applicable.
